# Privacy preservation in blockchain-based healthcare data sharing: A systematic review

**DOI:** 10.1007/s12083-025-02148-9

**Published:** 2025-10-17

**Authors:** Kun Li, Ankur Lohachab, Michel Dumontier, Visara Urovi

**Affiliations:** Institute of Data Science (IDS), Maastricht University, Minderbroedersberg 4-6, Maastricht, 6211 LK The Netherlands

**Keywords:** Privacy, Blockchain, Healthcare data sharing

## Abstract

Blockchain technology promises enhanced data ownership, control, and interoperability in healthcare, yet security and privacy concerns continue to hinder its adoption. Existing surveys examine blockchain-based privacy challenges, but they lack a systematic analysis and maturity evaluation of privacy-preserving techniques tailored to healthcare data sharing. This paper presents a systematic review of blockchain-based privacy-preserving solutions, analyzing blockchain details, applied privacy methods, regulatory compliance, and maturity levels using Technology Readiness Levels (TRLs). Our findings reveal that authentication and authorization is the most explored stage, dominated by smart contracts and ciphertext-policy attribute-based encryption. Proxy re-encryption is frequently used for data transfer, while privacy-preserving search and verification remain underexplored. On/off-chain mechanisms are commonly applied to balance privacy and storage efficiency. TRL assessment shows that most solutions remain at the proof-of-concept stage (TRL3), with only limited progress to prototype validation (TRL4–TRL5), highlighting the gap between experimental designs and real-world deployment. To guide developers and researchers, we identify two primary patterns of blockchain integration and propose a framework for system design. We also compare methods across data-sharing stages, outlining their strengths and limitations to support informed selection. In conclusion, while research interest is growing, the field remains at an early stage of maturity. Addressing this gap requires stronger implementation capacity, access to clinical data, and robust regulatory alignment. We emphasize the importance of clinical validation and real-world testing to advance privacy-preserving blockchain solutions toward practical adoption in healthcare.

## Introduction

Healthcare data are valuable for driving medical research and improving healthcare outcomes [1]. Sharing and analyzing healthcare data can improve healthcare services, accelerate biomedical discoveries, and reduce medical costs. However, despite these advantages, legal and privacy concerns have prompted institutions, such as hospitals, to silo their data, leading to fragmented data landscapes. This fragmentation poses significant barriers to leveraging cutting-edge technologies such as big data, artificial intelligence, and cloud computing and results in inefficiencies, single-point-of-failure vulnerabilities, and the risk of potential data breaches [2]. Furthermore, a key issue lies in patients’ limited control over their healthcare data, which hampers their access to personalized and tailored healthcare experiences.

These challenges are further exacerbated by the increasing frequency and severity of real-world data breaches, highlighting the vulnerabilities of centralized healthcare data systems. For instance, in May 2023, Tampa General Hospital in the U.S. suffered a data breach that exposed approximately 2.1 million patient records, resulting in a class-action settlement of $6.8 million, with individual payouts of up to $7,500 [3]. Similarly, in July 2025, AMEOS Group–a major European healthcare provider–disclosed a cyberattack that compromised sensitive information belonging to patients and employees across multiple countries [4].

Such incidents are not isolated. According to the HIPAA Journal, over 133 million patient records were compromised in 2023 alone across 725 reported breaches in the United States [5]. Regulatory bodies have also imposed steep penalties: under the GDPR, healthcare providers in Sweden and France have received fines of 3.2 million Euros (2024) and 1.5 million Euros (2023), respectively, for mishandling patient data [6, 7].

Together, these breaches and regulatory actions underscore the urgent need for robust, privacy-preserving data-sharing architectures. Traditional privacy-preserving techniques–such as encryption, access control, anonymization, differential privacy, and audit logging–provide important protections but remain insufficient for large-scale, cross-institutional healthcare data sharing (Table 1). Specifically, encryption protects content but leaves metadata vulnerable to traffic analysis and linkage [8–10]; access control is commonly centralized with limited cross-organizational auditability [11]; anonymization and pseudonymization are vulnerable to re-identification [12, 13]; differential privacy introduces utility trade-offs in health data [14]; and centralized audit logs are tamper-prone [15]. These limitations highlight the need for more robust and decentralized solutions. Blockchain, with its inherent properties of decentralization, immutability, and transparent auditability, offers a promising foundation to overcome these shortcomings.

**Table 1:** Traditional privacy-preserving techniques and their limitations in healthcare data sharing

Technique	Description	Limitations in Healthcare Data Sharing
Encryption	Symmetric/asymmetric encryption; protects data content from unauthorized access	Does not prevent metadata leakage, transaction linkability, or inference attacks; limited support for fine-grained access control
Access Control	Restricts access based on roles, attributes, or policies	Typically centralized, creating single points of failure; lacks transparency and auditability across institutions
Anonymization / Pseudonymization	Removes or substitutes identifiers (e.g., k-anonymity, pseudonyms)	Vulnerable to re-identification through data linkage or cross-referencing with external datasets
Differential Privacy	Adds statistical noise to query results or datasets	Difficult to apply to real-time or individual-level healthcare data; trade-off between privacy and utility
Audit Logging	Centralized logs of data access events	Logs may be altered or deleted; lack of tamper-resistance and limited cross-institutional accountability

Originating with Bitcoin [16] in 2008, blockchain is a decentralized database that possesses unique properties, including distribution (multiple parties transactional maintain the database), tamper-resistance (no one can change previous events or transactions), transparency (all parties have access to event records), and decentralization (there is not one party making all the decisions). Through the utilization of consensus mechanisms, blockchain networks achieve a unified and immutable state, ensuring the integrity of data. As a result, operations over sensitive healthcare data can be accurately tracked by transactions within the blockchain system, facilitating transparent and trustworthy data sharing. By connecting actors through a decentralized network, blockchain provides convenient data management, eliminating single-point-of-failure concerns. The inherent properties of blockchain hold significant potential in promoting healthcare data sharing while allowing patients to maintain control over who can access and utilize their data and for what purposes.

However, blockchain can also introduce privacy risks, such as identity inference attacks [17–19], transaction linkability attacks [10, 20], and smart contracts vulnerabilities [21]. All transaction data on the blockchain are transparent and immutable. This attribute can expose the identity of users by analyzing blockchain transaction graphs [17]. For instance, attackers can identify patterns that link specific healthcare interactions to real-world identities by analyzing blockchain transactions and cross-referencing them with external data (like social media or public records) [19]. Transaction linkability attacks also affect healthcare because they enable attackers to correlate multiple transactions, thereby reconstructing a patient’s medical history [22]. Malicious smart contracts further worsen these privacy risks. If a smart contract is not securely designed, it could be used to expose sensitive patient data or compromise the integrity of healthcare transactions. For example, a flawed smart contract could grant unauthorized access to medical records or leak private information during interactions between patients, healthcare providers, or insurers [23].

Besides, Healthcare data are subject to stringent regulatory frameworks aimed at safeguarding patient privacy and ensuring secure data handling [24–26]. In the United States, the Health Insurance Portability and Accountability Act (HIPAA) and the Health Information Technology for Economic and Clinical Health (HITECH) Act mandate strict control over patient data, setting standards for data confidentiality, integrity, and availability [24, 27]. In the European Union, the General Data Protection Regulation (GDPR) imposes strong requirements for data protection and grants individuals rights such as the “Right to Erasure,” data portability, and explicit consent management [25, 28]. Beyond these major regulations, other countries also enforce their own data protection laws, such as Australia’s Privacy Principles (APPs), each introducing unique compliance obligations and potential legal risks when sharing healthcare data across borders [26].

Although ongoing efforts strive to strike a balance between maintaining transaction auditability among participants and safeguarding user rights, such as ensuring the right to be forgotten, meeting these regulatory requirements presents additional complexity in blockchain-based healthcare solutions [24–26].

This paper presents a systematic review of privacy-preserving solutions for blockchain-based healthcare data sharing. Existing surveys have either focused on privacy in general blockchain systems [29–32] or on blockchain applications in healthcare without addressing technical privacy mechanisms [33–38]. A few works, such as [39–42], touch on privacy in healthcare but remain at a high level and lack technical depth.

Qi et al. [43] offer a comprehensive taxonomy of privacy threats and countermeasures and examine architectural choices and trade-offs, especially for IoT-driven medical environments. Furthermore, the rapid adoption of Healthcare Internet of Medical Things (IoT) technologies, including wearable sensors and remote monitoring devices, has raised unique privacy challenges [44]. These privacy challenges include large-scale data generation, the resource constraints of IoT devices that limit the use of computationally intensive cryptographic techniques, and the increased risk of data breaches due to highly distributed and heterogeneous system architectures [43, 45, 46]. Existing IoT-specific surveys, such as Khan et al. [45] and Alkhateeb et al. [46], provide valuable insights into security and privacy challenges in IoT-enabled healthcare systems but seldom address the integration of blockchain technologies. Our study complements these works by systematically analyzing privacy-preserving blockchain solutions that also address IoT-driven healthcare scenarios. Nevertheless, a key gap remains: these surveys lack an assessment of the maturity of existing solutions–an aspect that is essential for evaluating real-world applicability and readiness. Furthermore, there is still no structured framework for integrating blockchain technologies into healthcare, nor practical guidance for selecting appropriate privacy-preserving techniques. These gaps hinder the translation of theoretical advancements into scalable and secure real-world implementations.

To better substantiate the novelty and scope of our work, we compare it against several representative surveys that focus on either blockchain in healthcare or privacy-preserving techniques. Table 2 presents a structured comparison highlighting the differences in terms of scope, depth of privacy analysis, maturity evaluation, actionable frameworks and regulatory mappings.

**Table 2:** Comparison with recent survey works on privacy-preserving blockchain in healthcare

Study	Target Domain	Privacy Focus	Stage-wise Analysis	Maturity Evaluation	Design Patterns	Regulatory Mapping
[29]	Multi-domain	Broad and well-classified privacy dimensions	✗	✗	✗	✓
[31]	IoT-based blockchain systems	General review on privacy in IoT blockchain applications	✗	✗	✗	✗
[45]	Healthcare IoT	Authentication techniques in IoT-enabled systems	✗	✗	✗	✗
[46]	IoT (incl. Healthcare)	Hybrid blockchain integration in IoT systems	✗	✗	✗	✗
[33]	Healthcare	Mentions privacy concerns, but not focused on technical privacy-preserving solutions	✗	✗	✓	✗
[38]	General-purpose healthcare blockchain applications	Mentions privacy concerns, but not focused on technical privacy-preserving solutions	✗	✗	✗	✗
[42]	Internet of Medical Things	General overview	✗	✗	✓	✗
[43]	Healthcare IoT	In-depth, technique-level	✗	✗	✓	✓
This work	Healthcare data sharing (including IoT)	In-depth, technique-level	✓	✓	✓	✓

In contrast to the existing works, this paper presents a systematic review and analysis of the state-of-the-art privacy-preserving research based on blockchain in healthcare scenarios. The novelty of this work consists of technique-level analysis, maturity evaluation, and actionable design guidance in blockchain-based healthcare data sharing.

Our contributions are three-fold:

- In this paper, we propose a structured framework for developers and researchers aimed at enhancing the effectiveness and resilience of blockchain-based privacy-preserving solutions in healthcare.
- This paper outlines actionable guidelines for preventing privacy breaches in blockchain-based healthcare data sharing.
- In this paper, we provide insights on how blockchain-based privacy-preserving solutions can advance to higher mature stages and move closer to real-world deployment by evaluating the maturity of these solutions in healthcare using Technology Readiness Levels (TRL).

The remainder of the paper is organized as follows. In section 2, we provide basic background knowledge related to data sharing in healthcare, the blockchain technology, privacy preservation in healthcare and technology readiness levels. In section 3, we present the methodology of this systematic review. Section 4 presents the data analysis, including an overview of selected studies, an analysis of gathered data, and an evaluation of selected studies. The findings are discussed in section 5. Finally, section 6 concludes the survey and suggests future works.

## Background

### Data sharing in healthcare

Healthcare data sharing has distinctive features that differentiate it from data management practices in other domains, emphasizing the sector’s requirements and the sensitive nature of the data. These features include: 1) **Sensitivity** (Healthcare data demands exceptionally stringent privacy and security measures to prevent unauthorized access and breaches [47, 48]); 2) **Regulatory Compliance** (Healthcare data is subject to rigorous regulations, which impose compliance with specific operational standards that are typically more stringent than those required in other domains [49]); 3) **Accuracy and Reliability** (The accuracy and reliability of healthcare data directly influence diagnosis, treatment plans, and outcomes [35, 50–52]); 4) **Accessibility** (Healthcare data must be accessible to various stakeholders, including doctors, nurses, insurance companies, and sometimes the patients themselves [53, 54])

Healthcare data sharing can occur in two main forms: primary data sharing, which involves the direct provision of data by the original collector, typically for immediate clinical use, and secondary data sharing, which refers to the sharing of data that has been previously collected and processed, often repurposed for research, quality assurance, or public health initiatives [55, 56].

**Fig. 1:**
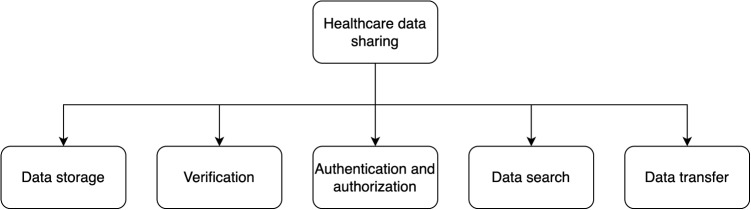
Primary stages in blockchain-based healthcare data sharing

To understand the processes involved in healthcare data sharing, it is helpful to consider the five stages: data storage, verification, authentication and authorization, search, and transfer [57–60], as shown in Fig. 1. A thorough evaluation of each stage is essential for understanding the complexities and challenges involved [61, 62].

**Data storage** involves securing healthcare data in repositories. It includes storing raw patient data, aggregated data, and the associated metadata [59]. In this stage, ensuring the integrity of stored data is important because any unauthorized changes or breaches can lead to serious legal, ethical, and medical consequences, particularly when data is accessed beyond the original clinical setting [58]. Modern healthcare storage solutions, such as cloud-based systems and distributed ledger technologies, must comply with regulatory standards (e.g., HIPAA and GDPR) to protect patient privacy and maintain data integrity, and compliant with secondary use requirements [63–65].

**Verification** involves confirming the integrity and authenticity of healthcare data to ensure it remains unaltered and reliable for further usage, such as research and public health analysis [66, 67]. In healthcare, maintaining data integrity and authenticity is essential because the insights derived from this data can influence healthcare policies, medical research, and public health decisions. Techniques such as cryptographic methods, digital signatures, and blockchain technologies are commonly used to maintain data integrity and authenticity [68]. This layer of verification supports the ethical and legal requirements of secondary data use by ensuring that the data remains as accurate and reliable as it was at the time of initial collection.

**Authentication and authorization** processes are fundamental to secure and compliant data sharing practices [69], particularly in healthcare [45]. Authentication refers to verifying the identity of users or entities attempting to gain access to a system. It ensures that individuals are who they claim to be and that only authorized entities can view or use the data for specific purposes, such as research or policy development [45]. Authorization, on the other hand, specifies the rights and permissions of authenticated users. It controls what actions are permitted, such as accessing de-identified datasets, viewing aggregated reports, or conducting specific analyses [69]. This stage is crucial for establishing controlled access and managed consent to sensitive healthcare data.

The **search** stage involves locating specific healthcare data that has been previously collected and stored for additional purposes, such as research or health policy analysis [70]. Efficient search capabilities are crucial in this context because timely access to relevant data can accelerate research outcomes and support data-driven healthcare improvements. Search mechanisms must be capable of handling both unstructured data (e.g., clinical notes, images) and structured data (e.g., lab results, diagnoses) in ways that maintain patient privacy and comply with regulatory standards [71].

**Data transfer** refers to the secure transmission of healthcare data between different systems, institutions, or stakeholders, such as research institutions, regulatory agencies, and healthcare providers [72]. Since these systems may adhere to various standards and protocols, robust security measures are essential to protect sensitive data during transit [73]. Techniques such as Proxy Re-Encryption (PRE) and Transport Layer Security (TLS) help ensure that data is not intercepted, tampered with, or exposed during transmission, thereby upholding privacy standards and compliance with regulatory frameworks [57].

### Blockchain

Blockchain is a decentralized ledger that ensures secure, transparent, and immutable transactions across distributed networks. Its key features–decentralization, immutability, and cryptographic security–make it particularly appealing for healthcare data sharing, where patient privacy and data integrity are paramount.

#### Blockchain types

Blockchains are commonly categorized into public, private, consortium, and hybrid types. Each offers distinct trade-offs in decentralization, performance, and access control. Table 3 summarizes their key characteristics and suitability for sensitive data sharing scenarios.

**Table 3:** Comparison of different blockchain types

Type	Access Model	Governance	Pros	Cons
Public (Permissionless)	Open to anyone (read, write, validate)	Community-driven (open-source)	Transparency, censorship-resistant	Scalability, privacy concerns, slower consensus
Private (Permissioned)	Restricted, single-entity control	Central admin(s)	High speed, privacy, compliance	Centralized trust, less transparency
Consortium	Restricted, multi-entity control	Collaborative among organizations	Shared trust, more efficient than public	Coordination overhead, partial transparency
Hybrid	Mix of public and private components	Varies; can be partially open	Flexible data access and privacy controls	Complex to implement and govern

As shown in Table 3, public blockchains prioritize decentralization and transparency but suffer from scalability issues. In contrast, private and consortium blockchains offer better performance and privacy but at the cost of decentralization. Hybrid blockchains attempt to balance these trade-offs, though their implementation remains complex.

#### Consensus protocol

Consensus protocols determine how blockchain networks achieve agreement on the next block. While various mechanisms exist, Proof of Work (PoW), Proof of Stake (PoS), and Practical Byzantine Fault Tolerance (PBFT) are among the most widely used. Table 4 compares their key characteristics, strengths, and limitations.

**Table 4:** Comparison of PoW, PoS, and PBFT

Feature	PoW	PoS	PBFT
Consensus Mechanism	Computational puzzle solving (mining)	Stake-based validator selection	Message-based agreement among nodes
Node Selection	Miners compete by solving cryptographic puzzles	Validators are chosen based on the amount of cryptocurrency staked	A predefined set of validators exchange messages to reach consensus
Energy Efficiency	Low (requires extensive computational power)	High (minimal computational work required)	High (no mining, relies on message passing)
Security	Secure against Sybil attacks [74] but vulnerable to 51% attacks [75]	Secure against Sybil attacks but may face centralization risks if few stakeholders dominate	Tolerates up to 1/3 of Byzantine nodes but requires honest majority
Scalability	Low (slow transaction processing, high energy cost)	Higher than PoW, but may depend on network design	Limited scalability due to communication overhead
Finality	Probabilistic (confirmations reduce risk of reversion)	Faster than PoW, but may require checkpoints	Immediate (deterministic finality, no forks)
Common Usage	Public blockchains (e.g., Bitcoin, Ethereum 1.0)	Public and private blockchains (e.g., Ethereum 2.0)	Permissioned blockchains (e.g., Hyperledger Fabric)

As shown in Table 4, each consensus mechanism presents unique trade-offs relevant to healthcare data sharing. PoW’s energy-intensive nature and low scalability make it impractical for healthcare applications requiring efficiency and environmental sustainability. PoS improves scalability and energy efficiency, making it a more viable option for public healthcare blockchains, though stake centralization risks remain. PBFT offers fast finality and high efficiency in permissioned environments, aligning well with private and consortium blockchains often favored in healthcare systems due to their need for access control and regulatory compliance.

### Privacy preservation in blockchain-based healthcare data sharing

Privacy refers to the ability to control and protect sensitive personal information from unauthorized access, disclosure, or misuse [76]. In healthcare, privacy is of paramount importance because patient data often includes highly sensitive information such as medical histories, diagnoses, treatments, and genetic information [77]. While blockchain technology provides an innovative approach to data sharing, it also presents unique privacy challenges in healthcare due to its inherent characteristics [33].

#### Privacy classification

To effectively protect privacy in a blockchain-based healthcare system, it is essential to recognize different types of privacy, including healthcare data privacy, identity privacy, and transaction privacy [32].

**Healthcare data privacy** involves protecting the actual content of healthcare data, such as medical records, diagnostic information, treatment history, and research results. It ensures that only authorized individuals or entities can view or modify sensitive health data [30, 32, 39]. In healthcare blockchain systems, **identity privacy** focuses on protecting the identities of the patients, healthcare providers, and other stakeholders involved. This aspect of privacy is essential to prevent attackers from linking transactions to real-world individuals through blockchain analysis [30, 39]. Furthermore, blockchain’s transparency makes **transaction privacy** a significant concern. This involves protecting the details of transactions, such as the participants, amounts, and transaction purpose, from unauthorized parties [32].

#### Privacy challenges

The three primary types of privacy, outlined in Section 2.3.1, are foundational to understanding the privacy landscape in blockchain-based healthcare systems. However, these types of privacy are confronted with challenges due to the inherent properties of blockchain technology.

The privacy challenges presented in blockchain-based healthcare data sharing include: 1) **Transparency and Immutability**. Blockchain’s transparency is beneficial for data integrity but creates risks when sensitive data is involved. Every participant in the network can access the transaction data, potentially exposing confidential patient information. Additionally, the immutability of blockchain records complicates compliance with privacy regulations that mandate the right to erase or correct personal data [37]. 2) **Identity Inference and Linkability**. In public blockchains, transactions are visible to all participants. This visibility can lead to identity inference, where attackers analyze transaction patterns to deduce the identities of participants. For instance, attackers may cross-reference blockchain data with external datasets to infer a patient’s identity or reconstruct their medical history [30, 39]. 3) **Vulnerabilities in Smart Contracts**. Smart contracts automate processes in blockchain networks, but poorly designed contracts can unintentionally expose patient data. Malicious smart contracts or those with security flaws could be exploited to gain unauthorized access to sensitive information or disrupt the integrity of healthcare transactions [32]. 4) **On/Off-chain Data Management**. Managing data between on-chain and off-chain storage is crucial for maintaining privacy while leveraging blockchain’s benefits. On-chain storage is transparent by design, which poses a privacy risk if sensitive information is stored directly on the blockchain. Off-chain storage can alleviate this but introduces complexities in ensuring data integrity and access control [24]. 5) **Privacy Regulation Compliance**. Healthcare data is subject to stringent privacy regulations worldwide. Inadequate privacy protections in blockchain systems may result in violations of regulations like GDPR and HIPAA. These regulations require data minimization, consent management, and the right to access, rectify, or delete data, which must be carefully addressed in blockchain-based solutions [63].

#### Privacy-preserving methods

Privacy-preserving methods in healthcare ensure that sensitive patient information remains secure and confidential while allowing for effective data analysis and collaboration among healthcare professionals. In this section, we summarize commonly used privacy-preserving methods in blockchain-based healthcare data sharing.

**On/Off-chain mechanism:** It is impractical to store the whole healthcare data on a blockchain [78]. Blockchain data is open to the public to some degree, which leads to challenges in preserving patients’ privacy. On the other hand, storing such a large amount of healthcare data on the blockchain can overwhelm the network and result in high prices for using this service [79]. Therefore, a common solution for the issue above is the on/off-chain mechanism: store the original healthcare data off the blockchain, for example, in a cloud or private data center, and the indices of these data on the blockchain. In this way, on-chain indices can reduce the cost using blockchain services, conceal the original data and facilitate data sharing. Off-chain data storage guarantees data ownership, privacy preservation, and less consumption of executing smart contracts.

##### Zero-knowledge proof

Zero-knowledge Proof (ZKP) [80] is an important technology in blockchain-based healthcare data sharing. For example, ZKP enables the patient (prover) to prove to a healthcare provider, researcher, or insurer (verifier) that they possess or satisfy certain conditions about their medical data–such as being the owner of certain medical records or having undergone specific medical tests–without revealing the actual data itself. For ZKP to function effectively in healthcare data sharing, three requirements must be met, as summarized in Table 5.

**Table 5:** Zero-knowledge proof requirements and their application in healthcare

Requirement	General Explanation	Explanation in Healthcare
Completeness	An honest prover can always convince an honest verifier if the statement is true.	If a patient (the prover) holds valid medical data, they should be able to convince the healthcare provider (the verifier) of the truth of the statement (e.g., possession of a medical record) without any ambiguity.
Soundness	A dishonest prover can convince an honest verifier with a small probability if the statement is false.	A dishonest patient should not be able to convince a healthcare provider of a false claim (e.g., pretending to own medical data that they do not possess).
Zero-knowledge	A verifier learns nothing except that the statement is true.	The healthcare provider learns nothing about the patient’s actual medical data except that the claim (e.g., the patient possesses the necessary medical record) is true.

##### Public-key encryption with keyword search

In healthcare, it is necessary to provide search functions that preserve privacy while sharing data. Public-key encryption with keyword search (PEKS) [81] can solve the problem of searching on data encrypted with a public key system. It is particularly useful when data is stored in the cloud or on a remote server and needs to be accessed by authorized users without knowing the decryption key. Using PEKS, this necessity can be met. While uploading data, a patient can publicly encrypt healthcare data by providing a key to identify some specific keywords; A proxy as a tester can classify the encrypted data and return the result while a user submits a requirement for some data without leaking the original data.

##### Smart contract

As computer programs, smart contracts [82, 83] were added to the blockchain by Ethereum [84] to build decentralized applications [85]. Other blockchain platforms also implement smart contracts, like chaincode in Hyperledger Fabric. In the context of healthcare, smart contracts play a significant role in facilitating secure and efficient data sharing by automating and enforcing pre-defined rules between patients, healthcare providers, insurers, and other stakeholders as shown in Table 6.

**Table 6:** Benefits of smart contracts on blockchain and their applications in healthcare data sharing

Benefit	Use cases	Benefits
Automation	In [86], the authors propose a blockchain-based Personal Health Record (PHR) architecture that employs smart contracts to implement multi-party authorization (MPA) and threshold cryptographic schemes to automate secure and trustable medical data sharing and access in PHR systems.	Automates the release of patient health records to healthcare providers, insurers, or researchers once patient consent is verified, reducing delays and manual interventions.
Precision	In [28], the individual’s consent over health data is represented precisely, and data requesters are enabled to search and access those data automatically	Ensures precise control over who can access specific healthcare data, for how long, and for what purpose, ensuring compliance with privacy regulations such as HIPAA or GDPR.
Transparency	In [87], the system described leverages blockchain and smart contracts for transparent access control and consent management for Electronic Health Records (EHRs). This process is transparent to all involved parties, ensuring auditable and tamper-resistant actions	Allows patients and healthcare providers to track who has accessed or requested access to medical records, increasing accountability and trust in the data-sharing process.
Persistence	In [71], fully functional access control is provided to ensure that patients and medical institutions can control access to the data	Guarantees that healthcare data-sharing agreements cannot be altered after deployment, ensuring the integrity and security of the terms agreed upon between patients and healthcare stakeholders.

##### Proxy re-encryption

In healthcare, original data is usually encrypted using data providers’ public keys and stored locally or in the cloud. Proxy re-encryption [88] is a primitive allowing a proxy to convert a ciphertext encoded under Alice’s (one participant) public key into one that can be decoded by Bob’s (another participant) private key. When a data requester applies for it, the proxy can transform the ciphertext of healthcare data into one that the requester can decode with their private key. This way, healthcare data can be shared without revealing individual privacy. With a combination of cloud computing and proxy re-encryption, blockchain-based healthcare data sharing solutions can solve the issue of storing and transferring large amounts of healthcare data.

### Technology readiness levels

To evaluate the maturity of technologies in blockchain-based healthcare data sharing, we adopt the Technology Readiness Levels (TRLs) framework [89] shown in Table 7.

**Table 7:** Overview of TRLs with corresponding EARTO definitions and application to healthcare technology evaluation

Cluster	TRL	EARTO reading	Our understanding
Invention	TRL1	Basic principles observed	Identification of basic principles that underpin the technology
	TRL2	Technology concept formulated	Conceptualization of how the technology could be used in a healthcare setting
Conceptvalidation	TRL3	First assessment of the feasibility of the concept and technologies with prototypes	initial feasibility assessed practically; Laboratory experiments with specific technology details
	TRL4	Validation of an integrated prototype in a laboratory	Integration of technology components tested in a controlled lab environment, simulating clinical conditions.
Prototyping and incubation	TRL5	Testing of the prototype in a user environment	Technology prototype tested in a relevant clinical environment, such as a pilot study in a hospital setting
Pilot production and demonstration	TRL6	Pre-production of the product, including testing in a user environment	Clinical trials or extensive testing demonstrating efficacy and safety in the intended healthcare environment
	TRL7	Low scale pilot production demonstrated	Large-scale validation through multicenter trials or studies, demonstrating clinical effectiveness and integration
Initial market introduction	TRL8	Manufacturing fully tested, validated and qualified	Technology has completed all regulatory reviews and is qualified for clinical use in real-world healthcare settings
Market expansion	TRL9	Production and product fully operational and competitive	Widespread clinical adoption and proven operational efficacy in diverse healthcare settings under normal operating conditions

Initially developed by NASA, TRLs provide a structured assessment tool widely adapted across industries to measure the readiness of technologies from conceptual stages to full commercial deployment [90]. According to the European Association of Research and Technology Organisations (EARTO), the TRL scale, shown in Table 7, is highly adaptable, reflecting its broad application from space exploration to healthcare innovations [91]. However, EARTO also highlights the need for adaptations to address specific sector challenges and the non-linear nature of innovation in contemporary R&D environments. This adaptability is essential in our study as it allows us to assess and categorize the technologies under review accurately, ensuring that stakeholders can clearly understand the readiness of each technology for implementation in real-world settings.

## Methodology

In this section, we describe in detail the workflow of our systematic review following the guideline by Keele, Staffs [92]. We introduce research questions, search strategy, and the inclusion and exclusion criteria for selecting the studies, followed by data gathering methods and data interpretation.

### Research questions

In line with our object to review privacy-preserving in blockchain-based solutions in healthcare data sharing, we aim to answer three research questions (RQs):

1. RQ1: How is blockchain technology applied to healthcare data sharing?
2. RQ2: Which privacy-preserving technologies are used in blockchain-based health data sharing solutions?
3. RQ3: To what extent privacy-preserving solutions based on blockchain are established in healthcare?

### Searching strategy

We selected peer-reviewed papers published in English from 2008 to 2024 with the following keywords: **privacy**, **healthcare**, **blockchain**, and **sharing**. We searched from the following engines and digital libraries: IEEE Xplore Digital Library, ScienceDirect, SpringerLink, ACM Digital Library, and Web of Science. The search terms and filters used in each digital library are enlisted in Appendix A.

### Eligibility criteria

To identify relevant papers, we defined inclusion criteria (IC) and exclusion criteria (EC) as shown in Table 8. The **Rationale** column presents a justification only when the relevance or importance might not be apparent from the criterion itself. Criteria that are self-explanatory based on their descriptions have been marked with a dash (“-") in the same column.

**Table 8:** Selection criteria for including studies in the systematic review of privacy-preserving solutions for blockchain-based healthcare data sharing

Selection criteria	Tag	Description	Rationale
Inclusion	IC1	Published in English between 2008 and 2024	-
	IC2	On the topic of preserving privacy based on blockchain in healthcare data sharing	Focuses review on the intersection of blockchain technology and privacy in healthcare
	IC3	Proposed and/or implemented a new approach or algorithm	-
	IC4	Applied existing approaches or algorithms to healthcare data sharing	-
	IC5	Designed and/or implemented a solution or architecture for healthcare data sharing based on blockchain	-
Exclusion	EC1	Publication type of survey, literature review, poster, workshop abstract, case study, discussion and grey literature	-
	EC2	Not considering healthcare as a topic, such as Internet of Things (IoT)	Critical to exclude as the review aims to address specific challenges and solutions relevant only to healthcare, ensuring focus and applicability.
	EC3	Not preserving privacy	-
	EC4	Not data sharing	Ensures the review remains focused on solutions that facilitate data exchange

### Overview of the review process

**Fig. 2:**
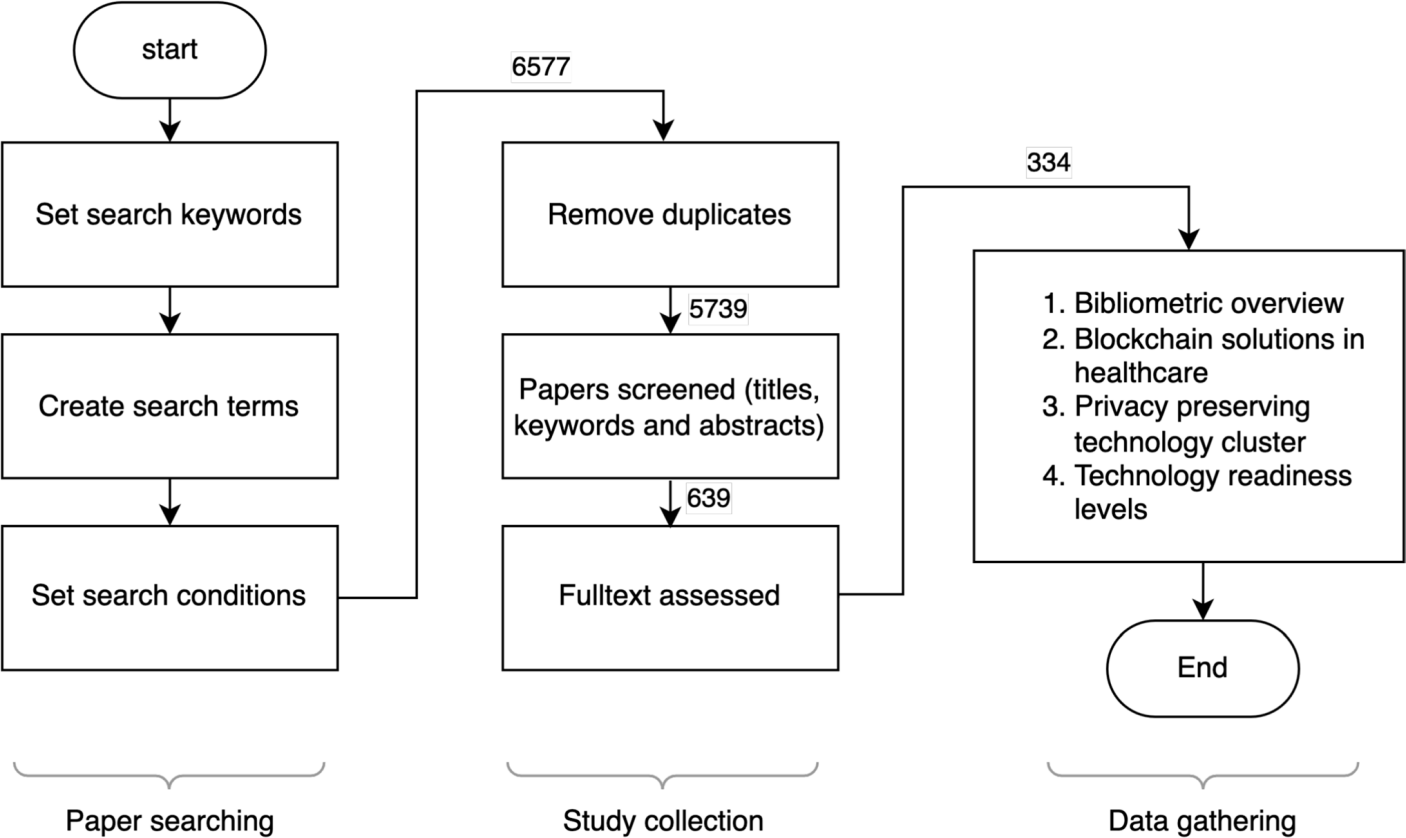
Methodology workflow for selecting relevant studies in the systematic review

Figure 2 shows the overall methodology steps used for selecting the relevant studies:

1. The search results were filtered according to the eligibility criteria listed in Section 3.3; In this step, we had the papers published in English from 2008 - 2024 (IC1), and the papers identified as relevant to our research questions by digital database searching engine(IC2), the result is presented in Table 9;
2. 5739 papers left after removing duplicates by Digital Object Identifiers (DOI);
3. Based on the relevance of the research questions (IC2), we evaluated and selected the results by checking the title, keywords, and abstract. In this step, we selected 639 papers using EC1-EC4;
4. The remaining papers were assessed in more detail by a full-text reading using IC3-IC5. In this step, we excluded papers with EC2-EC4. Finally, 335 papers were included in the review.
5. One paper was retracted during this study; the final number of selected papers was 334.

**Table 9:** Summary of search results

Data Sources	Papers identified and screened
IEEE Xplore	692
ScienceDirect	2472
SpringerLink	2119
ACM	758
Web of Science	536
Total	6577

**Fig. 3:**
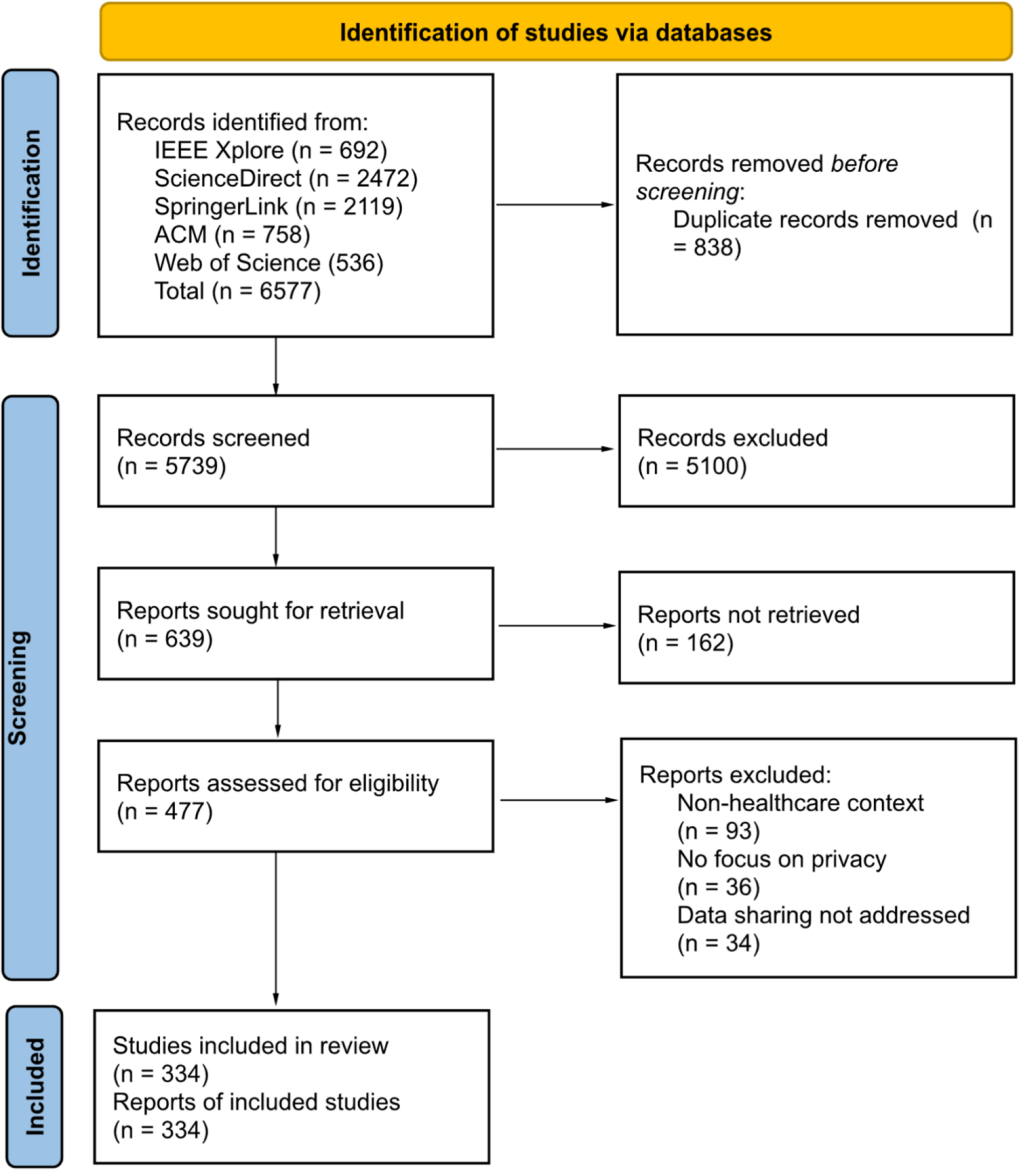
PRISMA 2020 flow diagram of study selection

As recommended by PRISMA 2020, we further illustrate the study selection process in a standardized flow diagram, as shown in Fig. 3.

### Data gathering

After selecting the relevant papers, we gathered data from 334 papers. For each paper, we initially collected basic information like title, author(s), and publication year. Furthermore, we focused on the specifics of the blockchain technology discussed in these papers, including consensus protocols, blockchain implementations, and types of blockchains. If a paper was missing the relevant information, we defined it as “Not discussed.” Additionally, we identified the privacy-preserving methods adopted in these solutions and the compliance assessment with regulations. A complete list of these studies, along with relevant details, is provided in Appendix B.

### Assessment of technology maturity using TRLs

In this study, we employ TRLs as a fundamental framework to assess the maturity of various privacy-preserving technologies involved in blockchain-based healthcare data sharing. TRLs provide a structured measure, from TRL1, indicating initial basic principles, to TRL9, representing a fully mature and market-ready technology.

Based on the EARTO reading and our understanding of the criteria for each level in Table 7, we developed a evaluation framework (Table 10), mapping observable evidence from reviewed studies to corresponding TRL levels. For example, Zhou et al. [93] introduced Med-PPPHIS, which is a blockchain-based solution validated in lab settings and preliminary applied in a real-world scenario (national physique monitoring in Anhui Province, China). According to Table 10, it was assessed at TRL5.

**Table 10:** The evaluation framework for TRL Assessment

TRL Level	Observable Evidence in Reviewed Studies
TRL1	Basic principles observed; conceptual discussion or theoretical analysis of blockchain use for healthcare data sharing.
TRL2	Technology concept and application formulated; initial design proposals without prototype or implementation details.
TRL3	Experimental proof-of-concept; preliminary implementation tested in isolated simulations or using synthetic datasets.
TRL4	Integration of technology components tested in a controlled laboratory environment, simulating clinical conditions.
TRL5	Technology prototype tested in a relevant clinical environment, such as a pilot study in a hospital or similar setting.
TRL6–TRL7	Pre-production and pilot deployment in real-world healthcare settings; extensive testing demonstrating efficacy and safety.
TRL8–TRL9	Full-scale production and deployment; regulatory approval and widespread clinical adoption in operational environments.

### Data interpretation and inference handling

In selected papers, some solutions did not provide enough information. For example, some theoretical solutions did not have a proof of concept, prototypes, or implementation, therefore lacking the information on blockchain details; other solutions briefly discussed technical details but missed some information. In order to get a better understanding of the technical details of blockchain solutions in healthcare, we made some deductions as follows:

- If the solution discussed the blockchain implementation but lacked the discussion on the consensus protocol, we would consider that it applied the default consensus algorithm. For example, Hyperledger Fabric^1^ solutions use PBFT as default during our literature review.
- If the solution discussed blockchain implementation but lacked a discussion on blockchain types, we would consider that blockchain type is similar to the common use cases. For example, although Ethereum can be deployed as a private permissioned blockchain, the most common scenario for applying it is as a public permissionless blockchain.

The use of imputation or inference to handle missing technical details in systematic reviews is not unprecedented. For example, reviews in meta-analysis frequently impute missing variance or outcome data to maintain comparability across studies [94, 95]. In our review, assigning PBFT to Hyperledger Fabric aligns with official documentation, which specifies PBFT as Fabric’s default consensus algorithm. Likewise, treating Ethereum implementations as public, permissionless blockchains reflects its current deployment scenario. These platform-specific defaults are not arbitrary choices but grounded in documented configurations and common usage patterns.

We recognize that such inference may introduce bias; however, it only affects a subset of the included studies (34.4% for consensus protocol and 14.4% for blockchain type). To ensure transparency, all inferred data points are fully documented in Appendix B. Moreover, sensitivity analysis shows that excluding inferred data does not materially alter the main findings (see Appendix B).

Our deduction is reasonable because:

- While conducting research based on blockchain with default configuration, researchers can focus on their research without considering specific blockchain elements;
- The common use cases of blockchain indicate a successful way of applying blockchain technology and provides enough experience of how to apply blockchain in healthcare.

Quantitatively, out of 334 studies, 205 (61.4%) did not report consensus protocols; of these, 125 (37.4%) were assigned inferred values. Similarly, 113 (33.8%) studies lacked information on blockchain types, and 75 (22.5%) were assigned inferred values based on the rules above.

To assess the robustness of our findings against the inference of missing data, we conducted a sensitivity analysis by recalculating the distributions after excluding studies with inferred consensus protocols (n=125) and, separately, studies with inferred blockchain types (n=75). The resulting distributions showed only minor changes. For consensus protocols, PBFT accounted for 27.9% of studies (vs. 26.9% in the full dataset) and PoW for 24.0% (vs. 30.5%). For blockchain types, public blockchains accounted for 22.6% of studies (vs. 24.6%) and consortium blockchains for 43.9% (vs. 41.9%).

These results suggest that the inference of missing data did not materially affect the observed distributions or the overall conclusions of this review. Full details are provided in Table 11.

**Table 11:** Sensitivity analysis of consensus protocol and blockchain type distributions

Category	Original (with inference)	Sensitivity (without inference)
Consensus Protocol		
PBFT	90 (26.9%)	36 (27.9%)
PoW	102 (30.5%)	31 (24.0%)
Blockchain Type		
Public	82 (24.6%)	50 (22.6%)
Consortium	140 (41.9%)	97 (43.9%)

## Results and analysis

This section summarizes the findings of our systematic review and provides an initial analysis. It focuses on the application of blockchain in healthcare, privacy-preserving technologies across different stages of data sharing, and the maturity evaluation of the reviewed solutions.

**Fig. 4:**
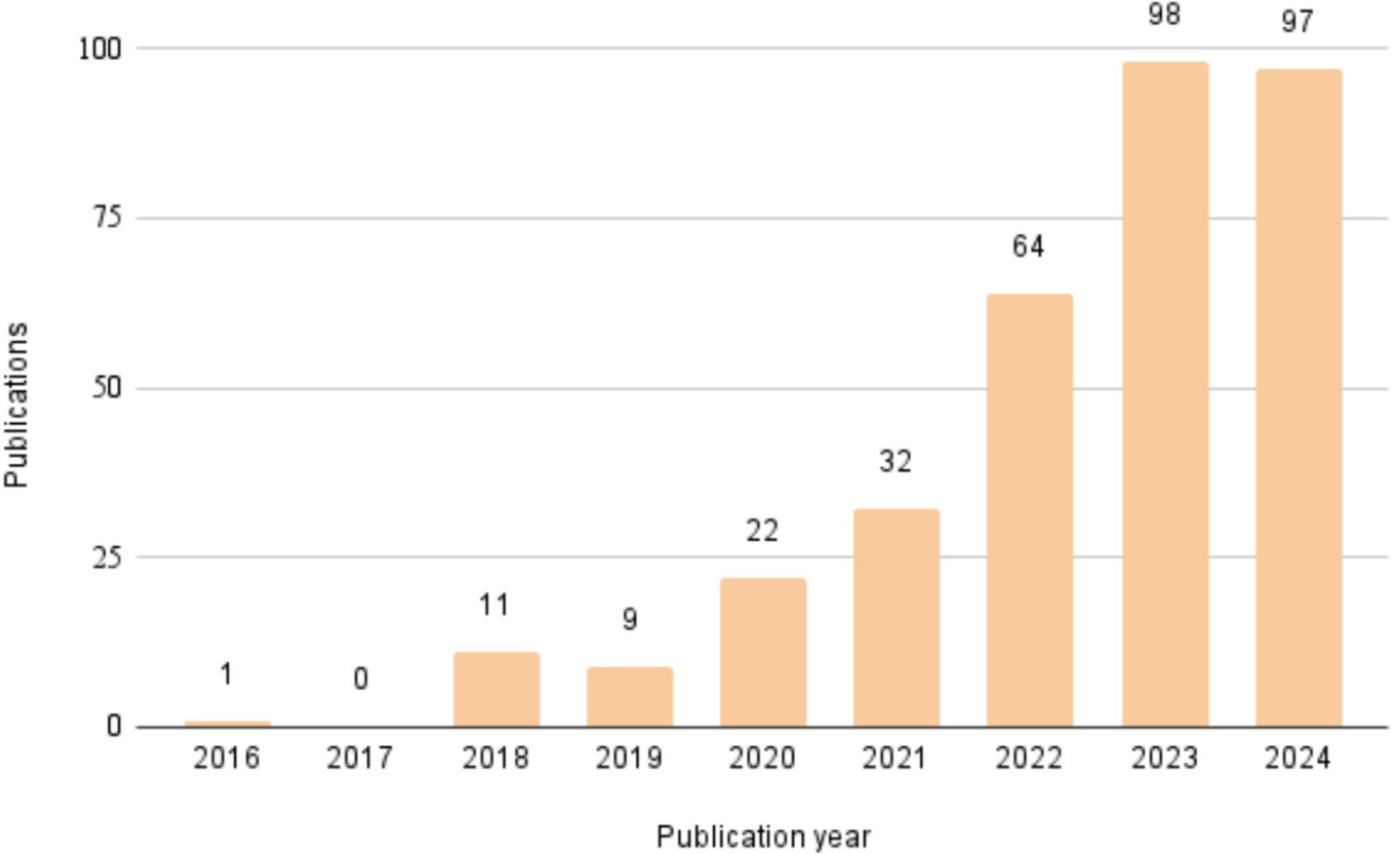
Annual publication trend of papers on blockchain-based privacy-preserving healthcare data sharing from 2016 to 2023

Figure 4 shows an overview of the selected papers based on the methodology described in Section 3. Over the span of nine years, there was limited research activity in the early years (2016-2019), with an significant increase from 2020 onward. This suggests that privacy-preserving blockchain-based solutions have become a highly active area of research in healthcare data sharing. A complete image of the extracted data in placed in Appendix B.

### Blockchain application patterns in privacy preserving healthcare data sharing

In this section, we provide an analysis of the selected solutions from the perspectives of blockchain types, blockchain implementations, and consensus protocols. Furthermore, we illustrate the application patterns observed in these solutions and figure out a framework for blockchain-based healthcare data sharing system design.

#### Blockchain types and implementations

**Fig. 5:**
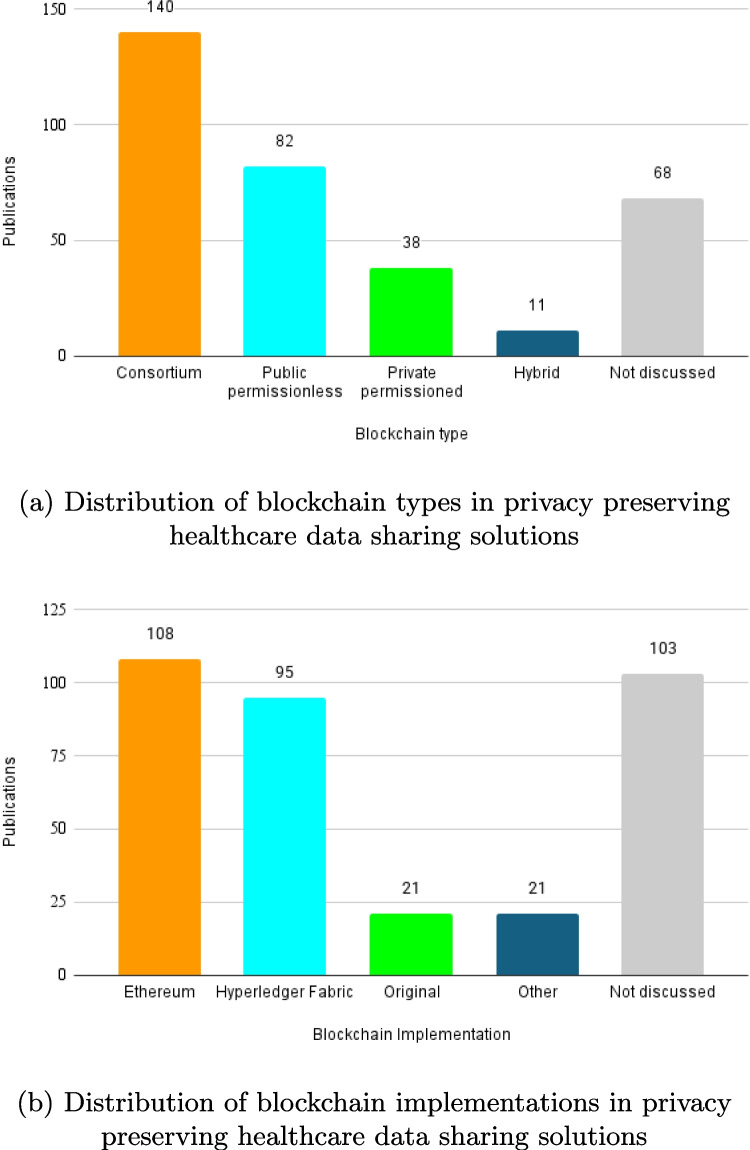
Blockchain type and implementation in privacy preserving healthcare data sharing solutions

Figure 5a shows that consortium blockchains are the most frequently used type in healthcare data sharing solutions, with 140 papers reporting their use. Public permissionless blockchains are the second most common, appearing in 82 studies. Private permissioned blockchains were utilized in 38 studies, while hybrid blockchains were mentioned in 11 studies. Additionally, 68 papers did not specify which type of blockchain was used.

Figure 5b presents which blockchain implementations were used. Ethereum is the most commonly used blockchain implementation, with 108 papers utilizing it. Hyperledger Fabric is the second most frequent, employed in 95 studies. Custom-built platforms were developed for healthcare use cases, were employed in 21 papers. In addition to widely used platforms like Ethereum and Hyperledger Fabric, 21 studies adopted less common blockchain platforms. These “Other” implementations are often experimental or customized for specific use cases, which offer specialized functionalities or performance optimizations. Additionally, 103 papers did not specify the blockchain implementation used.

#### Consensus protocols

**Fig. 6:**
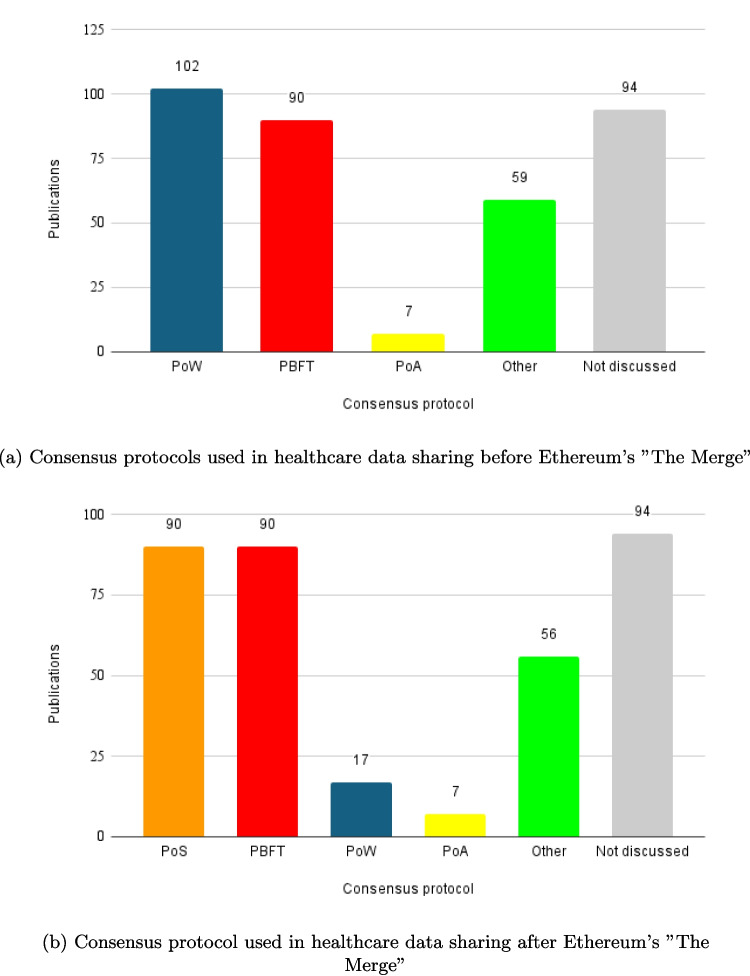
Comparison of consensus protocols used in blockchain-based privacy preserving healthcare data sharing solutions before and after Ethereum’s “The Merge”

Figure 6 presents the results of the consensus protocols employed in blockchain-based privacy preserving healthcare data-sharing solutions, distinguishing between those used before and after “The Merge,” which refers to Ethereum’s transition from Proof of Work (PoW) to Proof of Stake (PoS), marking a pivotal moment in blockchain technology [96].

**Before “The Merge”**, PoW was the most commonly used consensus mechanism employed in 102 papers. PBFT appeared in 90 papers. Proof of Authority (PoA) were used in 7 papers. The “Other” consensus protocols category comprises less frequently used mechanisms that were reported in 59 studies before “The Merge.” In total, 94 papers did not specify the consensus protocol used.

**After “The Merge”**, PBFT remained common, with 90 papers. PoS became the second most used consensus protocol, with 90 papers adopting it. PoW usage decreased to 17 papers. PoA was reported in 7 papers. Other consensus mechanisms were mentioned in 36 papers. Again, 94 papers did not specify the consensus protocol used.

#### Blockchain application patterns

As discussed in Section 1, applying blockchain to privacy preserving healthcare data sharing presents various challenges, and no single solution solves all of them. However, various blockchain-based solutions have been proposed, each addressing specific challenges. These solutions take into account three main factors–blockchain type, consensus protocol, and blockchain implementation–which should be considered together to create privacy-preserving and efficient solutions.

**Fig. 7:**
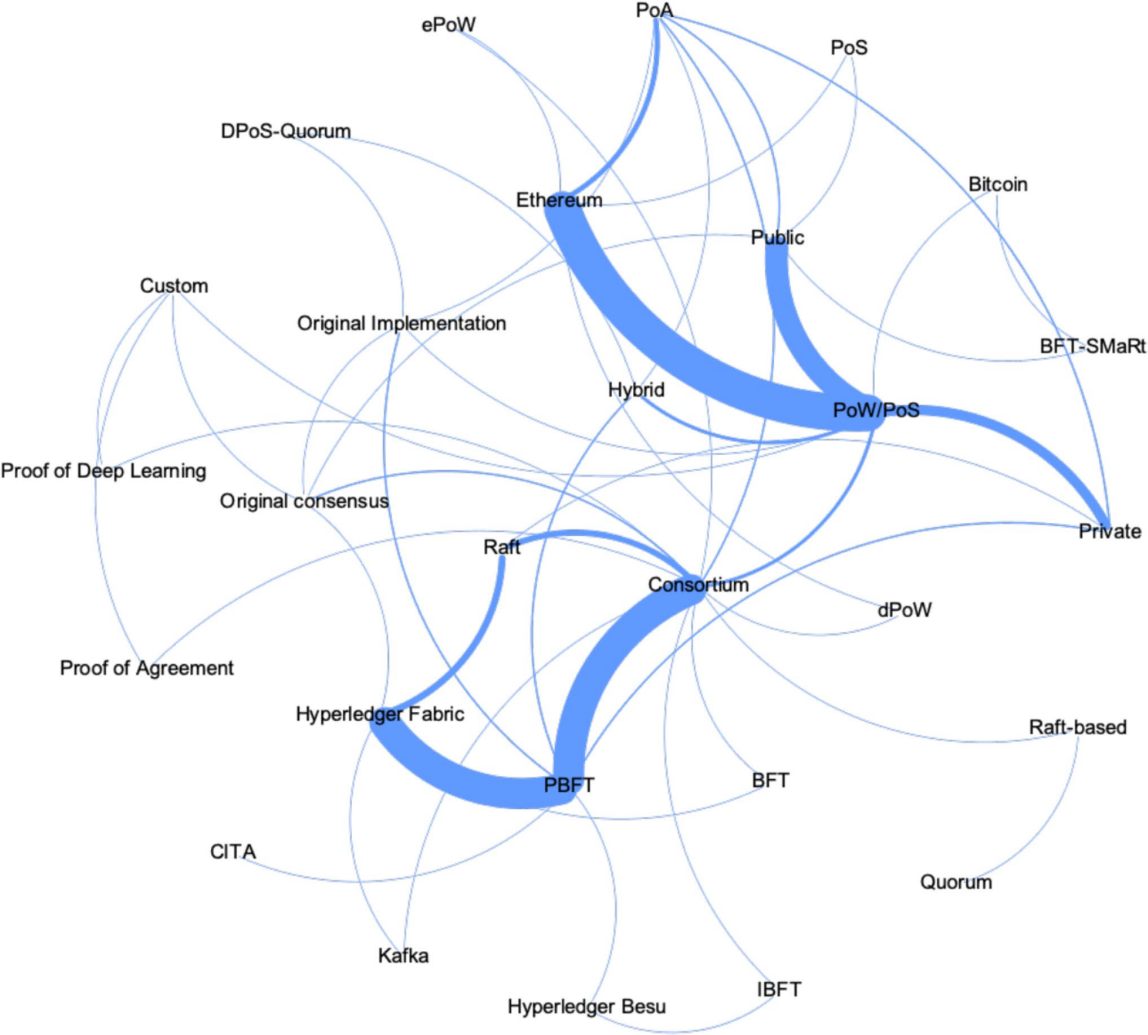
Application patterns of blockchain technology in privacy preserving healthcare data sharing solutions

Figure 7 presents these three factors for studies that provide information on all of them. In this figure, each node represents one of the three factors: a blockchain type, consensus protocol, or blockchain implementation. A special node, labeled PoW/PoS, is included to account for “The Merge” in Ethereum, as explained in Section 4.1.2. Curved lines between nodes indicate relationships between the factors, with the thickness of each line representing the number of solutions that connect the two nodes. For instance, the line between “Consortium” and “PBFT” is significantly thicker than the line between “Consortium” and “Raft” [97], indicating that more solutions use Consortium blockchains with PBFT consensus protocol than with Raft.

Two patterns of blockchain application in healthcare emerge from Fig. 7:

1. **Consortium blockchain pattern:** This pattern utilizes a consortium blockchain, with PBFT as the consensus protocol, and is implemented using Hyperledger fabric. In this pattern, the consortium blockchain provides greater privacy since participants can control who accesses the network. PBFT processes transactions efficiently, making it suitable for high-throughput applications. The Hyperledger fabric delivers a highly modular and configurable architecture as an enterprise-grade platform. This pattern considers that access control, performance, and cost of sharing healthcare data are preferred while losing decentralization to some extent. The properties and limitations of the consortium blockchain pattern, as outlined in the reviewed literature, are summarized in Table 12. This table highlights the benefits of scalability, permissioned access, performance benefits, and support for smart contracts of this pattern, along with related issues like reliance on trust and potential scalability. As shown in Table 12, the consortium blockchain pattern demonstrates strong advantages in high scalability, permissioned access, and high performance, making it well-suited for regulated healthcare environments. However, its reliance on trust among consortium members highlights a critical limitation of reducing decentralization, particularly in competitive or multi-stakeholder scenarios. This trade-off between performance and decentralization underscores the need for tailored blockchain solutions in healthcare data sharing.

**Table 12:** Properties and limitations for consortium blockchain pattern in privacy preserving healthcare data sharing solutions

Properties	Limitations	References
High scalability: The system can efficiently handle increasing amounts of transactions or data without performance degradation	May require substantial computational resources for scaling infrastructure.	[87, 98–104]
Permissioned access: Only authorized participants can join the network, ensuring controlled and secure data sharing within the consortium.	Relies on trust between consortium members, which may limit adoption in competitive environments.	[79, 87, 99, 101, 102, 105–109]
High performance: The consortium blockchain, in combination with the PBFT consensus algorithm, enables fast transaction throughput and efficient resource usage.	Performance may degrade as the number of nodes increases due to communication overhead.	[87, 98, 99, 101–103]
Support for smart contract: Smart contracts are supported, allowing automation of processes and enforcing predefined rules within the system.	Complex smart contracts can be challenging to develop and maintain, increasing operational risks.	[106, 110]
2. **Public permissionless blockchain pattern:** This pattern involves the use of a public, permissionless blockchain, leveraging PoW/PoS as the consensus protocol and built on Ethereum. This pattern attaches importance to openness and decentralization regardless of the efficiency and performance problems. As presented in Table 13, all solutions adopting this pattern in this review integrate smart contracts, with particular emphasis on Ethereum’s openness and decentralized nature, Table 13 summarizes the properties and limitations of public permissionless blockchain patterns as observed in privacy-preserving healthcare data sharing solutions. This pattern emphasizes decentralization and transparency but faces challenges related to scalability, transaction costs, and vulnerabilities in smart contracts. As outlined in Table 13, the public permissionless blockchain pattern promotes decentralization and openness, making it ideal for patient-centric applications. However, their high transaction costs during peak usage and the vulnerabilities in smart contracts pose significant challenges. These limitations highlight the need for optimization strategies to enhance their feasibility in large-scale healthcare data sharing.

**Table 13:** Properties and limitations for public permissionless blockchain pattern in privacy preserving healthcare data sharing solutions

Properties	Limitations	References
High openness and decentralization: An open, decentralized structure allows any participant to access and verify transactions, promoting transparency and trust.	Can result in high transaction costs and scalability issues, especially during peak usage periods.	[86, 111, 112, 112–114]
Support for smart contracts: Smart contracts are supported, allowing automation of processes and enforcing predefined rules within the system.	May introduce vulnerabilities if smart contracts are not securely implemented.	[27, 28, 68, 111, 115–125]

In addition to their properties and limitations, the alignment of the two primary application patterns with regulatory requirements is crucial for their adoption in healthcare. Table 14 summarizes their regulatory fit, associated challenges, and potential improvements. These patterns exhibit distinct strengths and limitations in navigating the complex landscape of healthcare regulations.

**Table 14:** Blockchain patterns and regulatory alignment

Blockchain Pattern	Regulatory Fit	Analysis
Consortium Blockchain	Better suited for compliance due to controlled access and permissioned structures.	Centralized control enables regulatory oversight but may raise concerns about trust among consortium members.
Public Permissionless Blockchain	Faces challenges due to transparency and immutability, which can conflict with GDPR and HIPAA.	Decentralized, requires additional privacy layers (e.g., ZKP, anonymization) to meet compliance.

As shown in Table 14, consortium blockchains are better suited for environments where regulatory oversight and controlled access are critical, such as hospital networks. However, their reliance on centralized control raises trust-related concerns, particularly among competing stakeholders. In contrast, public permissionless blockchains, though decentralized and transparent, face challenges in maintaining compliance with privacy-focused regulations like GDPR due to their immutable nature.

#### Framework for blockchain-based healthcare data sharing system design

Designing blockchain-based healthcare data-sharing systems poses significant challenges, including balancing privacy, scalability, and compliance with stringent regulatory standards. To address these challenges, we propose a framework that guides system design by aligning blockchain type, consensus protocol, and implementation from Sections 4.1.1–4.1.3. This framework provides actionable insights into configuring systems that meet healthcare-specific requirements.

1. **Blockchain Type**: The choice of blockchain type (consortium, public permissionless, or private permissioned) significantly impacts privacy and access control. Consortium blockchains are often favored in healthcare settings due to their ability to provide controlled access among trusted participants, ensuring compliance with stringent regulatory requirements [102]. In contrast, public permissionless blockchains are chosen for their inherent openness and decentralization, which make them well-suited for patient-centric applications and transparent data sharing across diverse stakeholders [111].
2. **Consensus Protocol**: The selection of consensus protocol (e.g., PBFT, PoS, or PoW) affects system efficiency, transaction throughput, and decentralization. For example, PBFT has been widely adopted in healthcare applications due to its ability to process transactions with high throughput and low latency. It is suitable for environments requiring frequent data exchanges, such as patient records sharing among multiple hospitals [126]. In contrast, PoS requires less computational energy and is increasingly used in public blockchain networks like Ethereum for broader, decentralized healthcare applications post-Ethereum Merge [117]. By selecting protocols that align with healthcare’s performance and regulatory needs, developers can better balance security with operational efficiency.
3. **Blockchain Implementation**: Choosing a specific blockchain platform, such as Hyperledger Fabric or Ethereum, directly influences the system’s modularity, security, and ability to support smart contracts. Hyperledger Fabric is particularly well-suited for healthcare consortia because of its modular architecture and support for permissioned networks. Research shows that Hyperledger Fabric allows for customizable access controls, enabling compliance with complex regulatory standards in healthcare settings [100]. Ethereum, on the other hand, with its robust smart contract capabilities, openness, and decentralization, is ideal for public, decentralized healthcare applications that require openness and user control, as demonstrated in Al Amin et al. [121].

Based on the components above and application patterns in Section 4.1.3, two primary design models are synthesized in Table 15, each tailored to specific requirements of healthcare data sharing systems. These models address different requirements of healthcare data-sharing systems, from clinical networks to patient-centric applications.

Table 15 outlines the two primary design models for blockchain-based healthcare data sharing systems. It highlights the specific features, benefits, and limitations of each model, providing actionable insights into their application in different healthcare scenarios.

**Table 15:** Framework for blockchain-based healthcare data sharing system design

Model	Description	Benefits & Limitations
Consortium Blockchain Model	Suitable for healthcare environments where data privacy, permissioned access, and high transaction throughput are prioritized. This model uses a consortium blockchain with PBFT consensus protocol, implemented on Hyperledger Fabric.	**Benefits:** Controlled access, high scalability, predefined rule enforcement through smart contracts. Better suited for compliance **Limitations: ** Less decentralization, depends on trust among participants
Public Permissionless Blockchain Model	Designed for systems emphasizing transparency and decentralization. This model employs a public, permissionless blockchain, typically on Ethereum with PoS consensus (post-Ethereum Merge). It is ideal for open-access, user-driven applications, such as patient-controlled records, providing decentralization and accessibility across institutions.	**Benefits:** Decentralization, transparency, suitable for user-controlled and open data sharing **Limitations:** Limited transaction throughput, potential energy inefficiency in large-scale usage. Faces challenges due to transparency and immutability, which can conflict with GDPR and HIPAA

As presented in Table 15, the consortium blockchain model is particularly well-suited for environments requiring stringent privacy controls and high transaction efficiency, such as hospital networks or clinical research collaborations. In contrast, the public permissionless blockchain model offers greater transparency and decentralization, making it ideal for patient-centered applications where user control is paramount. These design models provide a flexible framework for tailoring blockchain solutions to diverse healthcare data-sharing needs.

To demonstrate the application and effectiveness of the framework described in Table 15, we present case studies. These examples showcase how specific configurations have been successfully applied in healthcare settings.

1. **Consortium Blockchain in Clinical Networks**: MedHypChain [102] is a blockchain-based system that aligns with the Consortium Blockchain Model outlined in Table 15. It employs a consortium blockchain using Hyperledger Fabric and PBFT consensus, prioritizing permissioned access and high transaction throughput. Designed specifically for healthcare interoperability, MedHypChain supports patient-centered data sharing by implementing an identity-based broadcast group signcryption scheme that enhances confidentiality, traceability, and unforgeability of transactions. MedHypChain’s architecture, which includes patient-proposal and patient-prescription blockchains, ensures that both patient and medical server maintain distributed ledgers, enabling real-time data exchange with high transaction throughput and regulatory compliance. This application exemplifies how a consortium model combined with PBFT can fulfill high transaction demands in regulated environments, achieving a balance between security, privacy, and operational efficiency.
2. **Public Permissionless Blockchain for Patient-Centric Health Records**: The proposed system in Tanwar and Thakur [127] exemplifies the Public Permissionless Blockchain Model detailed in Table 15. This system utilizes Ethereum’s public blockchain with Proof of Stake (PoS) consensus to balance openness and privacy, making it well-suited for patient-centric applications. It introduces Soulbound Tokens (SBTs), which are non-transferable tokens that allow patients to retain ownership of their medical data. Using SBTs, patients can selectively grant and revoke access to their EHRs, ensuring data control and privacy. Furthermore, the framework employs the InterPlanetary File System (IPFS) to store encrypted health records, enhancing scalability and data integrity through decentralized storage. This approach showcases how a public blockchain like Ethereum can empower patients in data sharing while maintaining transparency and robust access management through smart contracts despite potential challenges in transaction costs during peak usage.

### Privacy preserving technologies in blockchain-based healthcare data sharing

In this section, we provides a comparative analysis of privacy-preserving technologies adopted in blockchain-based healthcare data sharing. We categorize the reviewed solutions by data-sharing stages and summarize their advantages, limitations, and application contexts using structured tables and synthesis.

**Fig. 8:**
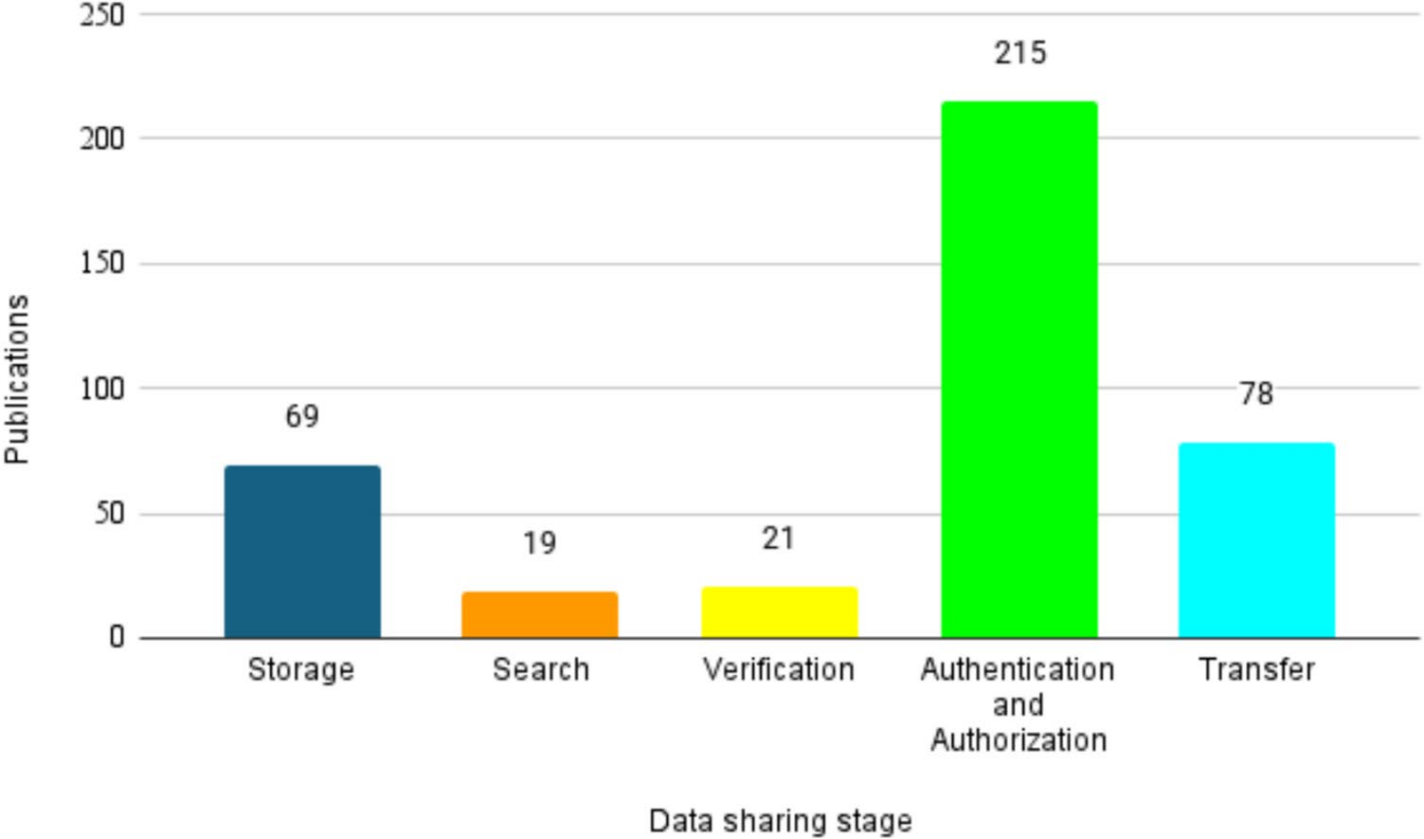
The distribution of studies dedicated to each stage of healthcare data sharing

We first present the overall distribution of research efforts across these five stages to identify which aspects of the data-sharing process have received the most attention. This provides the foundation for our subsequent stage-by-stage analysis. Figure 8 illustrates the distribution of studies focusing on different stages of the healthcare data-sharing process in blockchain-based privacy-preserving solutions. This figure categorizes 334 selected studies into five stages: Data Storage, Search, Verification, Authentication and Authorization, and Transfer.

The results reveal a significant concentration of research on the Authentication and Authorization stage, with 215 studies (64%) addressing this area, highlighting its importance for ensuring secure and privacy-preserving authentication and authorization mechanisms. The Data Transfer stage is the second most studied, with 78 papers (23%) focusing on secure transmission methods. Data Storage receives attention in 69 studies (20%), emphasizing on/off-chain storage designs to protect sensitive health data. Conversely, the Verification and Search stages are relatively underexplored, with only 21 (6%) and 19 (5%) studies each, suggesting potential gaps and opportunities for future research. These findings indicate a research trend where privacy preservation is predominantly tackled during access control (Authentication and Authorization), while upstream (Search, Verification) and downstream (Data Transfer) stages receive less attention.

#### Data storage

Data storage is a crucial component of blockchain-based healthcare data-sharing systems, involving both on-chain and off-chain storage to enable the secure storage of sensitive healthcare data while ensuring robust privacy protections. To achieve these goals, various privacy-preserving technologies are employed.

In the studies reviewed in this paper, some approaches address both on-chain and off-chain activities, using blockchain operations to manage metadata, access control, and verification while storing the actual data off-chain. On the other hand, other studies focus solely on data storage, where sensitive data remains on the blockchain. Consequently, these technologies are classified into two main categories: **On/Off-Chain Mechanisms** and **Encryption-Based On-Chain Methods**, each designed to address specific privacy requirements related to data storage.

**On/off-chain mechanisms**. By storing sensitive data off-chain while using the blockchain to manage metadata, access control, and transaction logs, on/off-chain mechanisms balance the privacy and scalability needs. In this study, we categorize these mechanisms into three patterns:

- **Off-chain storage/on-chain sharing**: This pattern keeps large or sensitive healthcare data in external storage (e.g., cloud repositories) while only hashes, pointers, or metadata are recorded on the blockchain. It reduces on-chain data load and privacy exposure but introduces potential risks if the off-chain environment is compromised. For example, in Miyachi and Mackey [24], the authors introduce a privacy-preserving blockchain framework that combines on-chain sharing and off-chain storage to enhance scalability, data privacy, and regulatory compliance for healthcare data management, enabling patient-centered control and secure data sharing.
- **off-chain storage/on-chain verification**: In this pattern, the actual data reside off-chain, and the blockchain serves primarily to verify data integrity or authenticity. While this can alleviate scalability and privacy concerns, the complexity of on-chain verification processes may add computational overhead and place greater trust in the off-chain repository. In Jiang et al. [128], the authors enhance privacy, authenticity, and throughput by combining off-chain storage with on-chain verification of the integrity and authenticity of electronic medical record and employing fairness-based transaction algorithms.
- **off-chain transfer/on-chain verification**: In this pattern, data are shared or transferred via an off-chain channel (e.g., Tor), and the blockchain records or confirms the validity of that transfer. This design enhances privacy by preventing direct on-chain exposure of sensitive data; however, it depends on a secure off-chain transfer mechanism to maintain end-to-end confidentiality. In Ali et al. [129], the authors propose a decentralized, privacy-preserving remote health monitoring system that leverages blockchain for secure identity management, authentication, and tamper-resistant record-keeping (on-chain verification) and uses Tor for private data delivery (off-chain transfer)

As summarized in Table 16, each pattern is designed to address privacy and scalability challenges while enabling secure and efficient data management, providing a flexible approach to balancing privacy, scalability, and integrity in healthcare data storage. Off-chain storage with on-chain sharing and off-chain transfers are particularly effective for reducing blockchain storage overhead. At the same time, on-chain verification methods enhance integrity without exposing sensitive information. However, these approaches rely on the security of off-chain systems, highlighting the importance of integrating robust access control and encryption techniques.

**Table 16:** The patterns of applying on/off-chain mechanisms in storing healthcare data

Pattern	Benefits	Limitations
Off-chain storage/on-chain sharing	- Sensitive records reside off-chain, so only references or metadata appear on the blockchain, reducing potential data exposure. - Storing large files off-chain diminishes blockchain bloat, enabling more efficient network performance.	Risks of metadata exposure and vulnerabilities related to access control and data breaches.
Off-chain storage/on-chain verification	- Healthcare data remain off-chain, protecting it from broad visibility on the public ledger. - The blockchain is less burdened since only proofs or integrity checks (not the raw data) are recorded.	Adds computational complexity for on-chain verification processes and depends on the integrity of off-chain storage systems.
Off-chain transfer/on-chain verification	- Actual data transfers occur through private or anonymous channels (e.g., Tor), shielding content from public view. - Data exchange happens independently of the blockchain, the network’s throughput is largely unaffected by large-volume transfers.	Requires trust in the off-chain transfer mechanisms.

**Encryption-based on-chain methods**. In blockchain-based healthcare systems, encryption is important for maintaining data confidentiality. Various encryption-based approaches are utilized to protect sensitive health records during storage. Based on our analysis, we identified the encryption methods in the literature and analyzed their benefits and limitations, as shown in Table 17.

**Table 17:** Encryption-based on-chain methods for storing healthcare data

Encryption Method	Description	Benefits and Limitations	References
Hybrid Encryption	Combines different encryption to balance security and efficiency.	**Benefits**: Combines features from different basic encryption methods, such as Advanced Encryption Standard (AES) and Elliptic-Curve Cryptography (ECC)**Limitations**: Vulnerability if one component encryption scheme is compromised	[130, 131]
Homomorphic Encryption	Enables computations on encrypted data without decryption, preserving data privacy in processing environments.	**Benefits**: Allows computations on encrypted data without decryption, ensuring privacy during processing.**Limitations**: Computationally intensive and slower compared to traditional encryption methods.	[132, 133]
Pairing-Based Cryptography	Uses bilinear pairings for secure key exchange and privacy-preserving data sharing in cloud and blockchain environments.	**Benefits**: Provides secure and privacy-preserving sharing of healthcare data in cloud and blockchain environments.**Limitations**: Computational overhead due to pairing operations.	[134]

As summarized in Table 17, encryption-based methods play a pivotal role in securing healthcare data on the blockchain. While hybrid encryption schemes offer flexibility and robust security, they remain vulnerable if a component encryption method is compromised. Homomorphic encryption is particularly advantageous for privacy-preserving data analytics, though its high computational cost may limit its applicability in real-time scenarios. Pairing-based cryptography, despite its computational overhead, ensures secure data sharing, especially in cloud-based healthcare systems. These methods underscore the trade-offs between privacy, computational efficiency, and scalability in blockchain-based solutions.

As stated above, some approaches address on-chain and off-chain activities, while others only concern on-chain storage. Both of them have reasonable functionalities and trade-offs, as the comparison shown in Table 18.

**Table 18:** Comparison of off-chain storage only vs. on-chain/off-chain mechanisms

Aspect	On-Chain Storage Only	On-Chain/Off-Chain Mechanisms
Definition	Encrypted data is stored on the blockchain.	Combines off-chain storage for data with on-chain components for verification, indexing, or access control.
Data Privacy	Only depends on the encryption mechanism	Balanced privacy with traceability; sensitive data remains off-chain, but references or metadata are stored on-chain.
Scalability	Low scalability; The sheer volume of data could make the blockchain practically unusable	Moderate scalability; blockchain usage for references or verification may introduce some overhead.
Data Integrity	High integrity; Blockchain’s inherent properties ensure tamper resistance and traceability	Enhanced integrity through blockchain-based verification (e.g., hashes, cryptographic proofs).
Transparency	High transparency	Moderate transparency; verifiable on-chain logs enhance accountability.
Regulatory Compliance	Conflicts with key regulatory requirements like the GDPR’s “right to be forgotten” and data sovereignty laws.	Well-suited for audit-heavy environments requiring verifiable integrity and compliance.
Implementation Complexity	More complex due to data management, scalability issues, regulatory compliance, and security challenges.	Moderate complex, requiring integration of blockchain and off-chain systems.
Scenarios	High-integrity audit-intensive systems; Healthcare fraud prevention.	Scenarios requiring data traceability and integrity verification (e.g., clinical trials, patient consent).

As illustrated in Table 18, on-chain storage offers robust data integrity and transparency but faces significant challenges in scalability and regulatory compliance. In contrast, on-chain/off-chain mechanisms balance privacy and scalability, making them well-suited for scenarios requiring traceability and auditability. However, their successful implementation relies on the secure integration of off-chain systems and robust cryptographic techniques

#### Search

Privacy-preserving search technologies play a crucial role in enabling secure access to healthcare data while maintaining patients’ privacy. These technologies are designed to address challenges posed by the sensitive nature of medical information, ensuring that data remains protected throughout the search process.

Among the limited studies addressing privacy concerns during the search process, three studies employed Public Key Encryption with Keyword Search (PEKS) [70, 135, 136]. By integrating the blockchain’s decentralized ledger with the privacy guarantees provided by PEKS, these approaches enable data providers to securely store encrypted data while maintaining search capabilities. In addition, other studies implemented searchable encryption schemes, each emphasizing different aspects of privacy and search efficiency [71, 137–141]. Table 19 provides a detailed summary of identified methods, highlighting their specific methodologies, benefits, and limitations.

**Table 19:** Summary of privacy-preserving search technologies in blockchain-based healthcare data sharing

Technology	Benefits and Limitations	Use cases
PEKS: Enables secure keyword searches on encrypted medical data.	**Benefits**: Ensures privacy during searches.**Limitations**: Computational overhead increases with keyword complexity	Retrieve specific diagnosis records from encrypted databases without exposing sensitive data [135].
Searchable Encryption: Enables searching directly on encrypted data without decrypting it.	**Benefits**: Maintains data confidentiality and supports efficient search operations.**Limitations**: Relatively high computational cost compared to traditional searches	Query encrypted EHRs for specific treatment histories or conditions while maintaining data confidentiality [139].
CP-ABSE: Combines Ciphertext-Policy Attribute-Based Searchable Encryption (CP-ABSE) with consortium blockchain.	**Benefits**: Provides fine-grained access control and reliable data search capabilities. **Limitations**: Complexity in managing attributes and policies can limit scalability	Search genomic datasets using attribute-based access control, ensuring compliance with data-sharing agreements [142].
MOPE: Utilizes Modular Order-Preserving Encryption (MOPE) for maintaining data order in range queries.	**Benefits**: Ensures privacy by encrypting sensitive data while allowing range-based queries.**Limitations**: Incompatible with the existing renowned blockchain platforms.	Identify patients within specific age groups or monitor disease progression trends securely [143].

As shown in Table 19, various privacy-preserving search technologies offer unique capabilities for ensuring data confidentiality and search efficiency. PEKS and searchable encryption provide robust guarantees on data confidentiality, though their computational demands can be a bottleneck in large-scale deployments. CP-ABSE introduces fine-grained access control while conducting confidential searches, making it ideal for multi-stakeholder environments, but its complexity may hinder scalability. MOPE addresses the need for range queries, improving search efficiency while maintaining privacy; however, it lacks compatibility with common blockchain platforms.

#### Verification

Verification in privacy-preserving healthcare data sharing based on blockchain is essential to ensure that data is accurate, authentic, and reliable. The reviewed studies address verification with a focus on three distinct areas: User Identity, Data Authenticity, and Data Integrity. These areas reflect the key requirements for building trust in blockchain-based systems while preserving privacy.

- **User Identity**: User identity verification ensures that users accessing the system are legitimate, without exposing personal details. This is achieved using technologies like ZKP and Attribute-Based Signatures, which enable fine-grained identity checks while maintaining privacy.
- **Data Authenticity** Data authenticity verification confirms that healthcare data originates from trusted sources and has not been altered. Technologies like Local Verifiability, Decentralized Attribute-Based Signatures (DABS), and Keyless Signature Infrastructure (KSI) play a vital role in achieving this goal by providing tamper-proof and decentralized methods of verification.
- **Data Integrity** Data integrity focuses on maintaining data completeness and ensuring that data remains accurate and trustworthy. Homomorphic Verification and ZKP are often employed to verify the integrity of encrypted data without compromising privacy, ensuring that sensitive information remains secure during verification processes.

As demonstrated in Table 20, verification technologies play a crucial role in ensuring data accuracy and reliability in blockchain-based healthcare systems. Solutions such as ZKPs and DABS provide privacy-preserving identity verification, while Local Verifiability and Homomorphic Verification ensure data authenticity and integrity. Despite their benefits, these technologies face challenges such as computational overhead and implementation complexity, emphasizing the need for optimized approaches in practical deployments.

**Table 20:** Verification stage solutions categorized by verification focus

Verifica- tion	Technology	Benefits and limitations	Use cases
User Identity	ZKP	Ensures privacy by verifying identity without exposing personal details. However, computational overhead may impact system performance.	In [144], ZKP is used with non-fungible tokens (NFT) to authenticate patient identities without revealing sensitive information.
	Attribute-Based Signature	Enables fine-grained control over identity verification based on attributes. However, attribute management complexity increases with larger datasets.	In [145], the verifier checks whether the attribute set of the patient satisfies the predicate to validate identity.
Data Authenticity	LocalVerifiability	Provides independent verification of individual data pieces without relying on centralized systems but requires auxiliary message generation, which adds complexity.	In [146], Local verifiability enables validation of individual pieces of health data using auxiliary messages generated by the patient.
	DABS	Maintains privacy while verifying the origin of data and signer attributes but has a higher computational cost for decentralized setups.	In [147], DABS ensures privacy-preserving verification of EHRs and signer identity based on attributes.
	KSI	Eliminates the need for key management but may have limited adoption due to specialized infrastructure requirements.	In [148], KSI ensures data authenticity without traditional cryptographic keys.
	Threshold Signature	Enhances trust by involving multiple authorities in the verification process but involves coordination overhead among authorities.	In [149], Multiple authorities sign user data submissions to guarantee privacy and data authenticity.
Data Integrity	Homomorphic Verification	Preserves data privacy while enabling third-party integrity verification. However, high computational complexity can limit scalability.	In [150], Homomorphic verification allows third-party auditors to verify the integrity of encrypted health data without decrypting the original data.
	ZKP	Protects sensitive information while ensuring data integrity verification but requires significant computational resources and expertise for implementation.	In [151], ZKP allows verification of encrypted data by various entities (patients, doctors, etc.) without disclosing sensitive information.

#### Authentication and authorization

As presented in Fig. 8, the authentication and authorization stage is noticeable as a research focus within the healthcare data sharing process, drawing interest in 215 studies. This stage involves two key scenarios: Access Control and Consent Management. Table 21 compares these scenarios in terms of objectives, key technologies, implementation complexity, and typical scenarios.

**Table 21:** Comparison of access control and consent management in authentication and authorization

Aspect	Access Control	Consent Management
Objective	Limit access to authorized entities based on roles or attributes.	Enable patients to dynamically manage permissions for their data.
Key Technologies	CP-ABE, RBAC using Smart Contracts.	Smart Contracts.
Implementation Complexity	Moderate: Requires defining roles and access policies.	Higher: Requires dynamic updates and user-friendly interfaces.
Scenarios	Role-based or attribute-based access to sensitive data, e.g., hospital systems.	Patient-centered data-sharing applications, e.g., research or clinical trials.

As illustrated in Table 21, Access Control mechanisms primarily focus on restricting data access to authorized entities through predefined policies, making them suitable for institutional use, such as hospital systems. On the other hand, Consent Management provides a patient-centric approach, empowering patients to dynamically grant or revoke permissions for their data. While Access Control offers streamlined implementation, Consent Management requires more complex designs to accommodate dynamic interactions and user-friendly interfaces.

These solutions predominantly focus on two main approaches: smart contracts and ciphertext-policy attribute-based encryption (CP-ABE).

**Smart contract-based solutions:** The use of smart contracts signifies a paradigm shift towards more autonomous and secure data sharing practices. Specifically, smart contracts serve as an automated intermediary that facilitates two main application patterns (Access Control and Consent Management) within the authentication and authorization stage. Each pattern offers unique benefits and addresses specific challenges in healthcare data sharing systems, summarised in Table 22.

- **Access Control:** Access control through smart contracts allows for the automatic enforcement of predefined rules, thereby ensuring that healthcare data is accessed only by authorized entities. For example, the authors in Dagher et al. [27] proposed a blockchain-based EHR management framework that empowers patients with the ownership and absolute control of EHRs and finally preserves patients’ privacy. In this framework, the authors create an ownership contract for tracking the records and a permissions contract that is specific to every record and stores the level of access (e.g., read, transfer, owner, or blind).
- **Consent Management:** Consent management facilitates a more dynamic and patient-centric approach, enabling patients to manage access permissions to their data directly. In Jaiman and Urovi [28], the authors enable data providers to decide how to share their data by introducing a dynamic consent model using blockchain, which represents and standardizes patients’ consent and matches it to the purpose statements provided by data users. With this solution, patients specify data sharing rules, monitor the use of data, and revoke or update access to the data.

**Table 22:** The patterns of applying smart contract in authentication and authorization

Pattern	Method	Benefits and Limitations	References
Access Control	Smart contracts autonomously enforce access rules upon certain conditions being met	**Benefits**: Enhances security and privacy by ensuring only authorized access. **Limitations**: The complexity of defining and managing access policies may lead to misconfigurations, potentially causing unauthorized access or denial of legitimate access.	[27, 71, 103, 114, 125, 126, 152–168]
Consent Management	Smart contracts enable patients to grant or revoke data access consent dynamically	**Benefits**: Provides patients with direct control over their data sharing preferences. **Limitations**: The dynamic nature of consent updates can increase the risk of delays or errors in managing access, particularly in systems with high transaction loads or poorly designed interfaces.	[28, 86, 87, 124, 169–171]

As shown in Table 22, smart contracts in access control autonomously enforce predefined rules, ensuring only authorized entities access sensitive data. However, the complexity of managing these policies can lead to potential misconfigurations, which necessitates robust policy design. Similarly, smart contracts facilitate dynamic consent management, empowering patients to control their data-sharing preferences in real-time. This approach aligns well with patient-centric applications, although it may introduce risks of delays in high-load systems.

**CP-ABE-based solutions**: CP-ABE is primarily leveraged to ensure fine-grained access control over sensitive healthcare data, where data owners define access policies based on attributes. Table 23 categorizes CP-ABE solutions into standard implementations and improved variants, highlighting their respective methods, benefits, and limitations.

**Table 23:** Summary of CP-ABE solutions in authentication and authorization

Category	Method	Benefits and Limitations	References
Standard CP-ABE	Enforces fine-grained access control based on user attributes	**Benefits**: Provides flexibility in defining access policies and controlling access to healthcare data securely. **Limitations**: High computational overhead, especially for decryption, and challenges in managing attributes for dynamic healthcare environments.	[142, 150, 172–179]
Improved CP-ABE	Optimized CP-ABE for edge computing and enhanced security	**Benefits**: Reduces computational costs for decryption, enhances scalability and performance in resource-constrained environments. **Limitations**: Increased complexity in implementation and potential compatibility issues with existing blockchain platforms.	[180, 181]

As summarized in Table 23, standard CP-ABE solutions offer flexibility in defining access policies, making them suitable for secure healthcare data sharing. However, their high computational overhead, particularly during decryption, poses challenges in dynamic and resource-constrained environments. To address these limitations, improved CP-ABE solutions introduce optimizations such as reduced computational costs and enhanced scalability. These improvements are particularly beneficial in systems with constrained resources, although at the cost of increased implementation complexity.

#### Transfer

The data transfer stage is a critical component of blockchain-based healthcare data sharing, as it involves securely transmitting sensitive patient information across systems while maintaining data privacy and integrity. Various privacy-preserving techniques have been identified in this study, as shown in Table 24. Below, we provide an overview of these techniques and their practical applications:

**Table 24:** Privacy-preserving techniques in the data transfer stage of blockchain-based healthcare data sharing

Technique	Benefits and Limitations	Use cases
Proxy Re-Encryption	**Benefits:** Enables secure delegation of decryption rights and maintains privacy. Scalable for multi-user systems. **Limitations:** Requires trust in the proxy and additional computational overhead for encryption transformations.	In [177], the authors present a blockchain-based vaccine passport verification system that leverages PRE to allow authorized parties to securely access encrypted vaccine data without involving the data owner, enhancing scalability, privacy, and compliance with data regulations
Homomorphic Encryption	**Benefits:** Allows secure computations on encrypted data without compromising privacy. **Limitations:** High computational cost and significant resource requirements make it challenging for real-time applications.	In [182], the authors propose a privacy-preserving federated learning framework that employs homomorphic encryption to secure the transfer and sharing of model parameters in healthcare data processing
Differential Privacy	**Benefits:** Prevents re-identification in aggregated data, ensuring anonymity and compliance with privacy regulations. **Limitations:** Introduces data accuracy loss due to added noise, requiring careful tuning of privacy parameters.	In [183], the authors introduce a decentralized machine learning framework for collaborative disease diagnosis that combines blockchain with differential privacy to secure gradient transfers, pseudo-identity for anonymity, and gradient delay compensation for asynchronous updates, ensuring privacy-preserving and efficient model training across institutions

##### Proxy re-encryption

Emerged as the most commonly used technique in the data transfer stage, Proxy Re-Encryption was implemented in 29 papers. By converting ciphertext encrypted under one user’s public key into ciphertext readable by another’s private key, proxy re-encryption ensures that access remains fine-grained and privacy-preserving. This method enables the secure delegation of decryption rights without exposing the underlying data, making it particularly effective for decentralized, scalable scenarios requiring fine-grained access control in healthcare systems.

##### Homomorphic encryption

Employed in 9 papers. The homomorphic encryption technique allows computations to be performed on encrypted data without decrypting it first, ensuring privacy throughout the data processing pipeline. Its ability to process encrypted data makes it invaluable for collaborative healthcare projects, such as multi-institutional studies or federated learning systems. Despite its computational intensity, homomorphic encryption is gaining traction for tasks like predictive modeling and personalized medicine.

##### Differential privacy

Used in 6 papers, differential privacy addresses the risk of re-identification in shared datasets by introducing statistical noise to data before it is shared. This technique ensures that while aggregated healthcare data can be used for research and analysis, individual patient records remain anonymous. It is particularly effective in large-scale public health initiatives where trends and insights need to be extracted from sensitive data without compromising individual privacy.

#### Comparative analysis of privacy-preserving methods

While the preceding Sections (4.2.1–4.2.5) presented privacy-preserving methods stage by stage, it is equally important to compare these methods holistically across key system requirements such as scalability, latency, and compliance. This comparative view highlights the inherent trade-offs of different techniques and provides guidance for selecting suitable methods in blockchain-based healthcare scenarios. Table 25 summarizes these trade-offs for representative methods.

**Table 25:** Comparative analysis of privacy-preserving methods in blockchain-based healthcare data sharing

Method	Scalability	Latency	Compliance
Zero-Knowledge Proofs (ZKP)	Moderate: proof size grows logarithmically, but trusted setup may limit scalability	High proof generation cost; verification relatively efficient	Enhances GDPR/HIPAA compliance via selective disclosure and consent enforcement
Homomorphic Encryption (HE)	Low–Moderate: computationally expensive, limiting real-time scalability	Very high due to expensive operations on ciphertexts	Enables privacy-preserving analytics, but may conflict with GDPR’s “right to be forgotten”
Differential Privacy (DP)	High: noise addition scales efficiently to large datasets	Low, since noise injection is computationally inexpensive	Strong alignment with GDPR principles (data minimization, anonymization)
Proxy Re-Encryption (PRE)	High: scales well in multi-user delegation scenarios	Moderate: requires proxy computation for re-encryption	Compliance achievable if proxy is trusted and actions are auditable
Ciphertext-Policy Attribute-Based Encryption (CP-ABE)	Moderate: fine-grained policies supported, but complexity increases with attributes	High decryption latency, especially in constrained devices	Supports regulatory compliance by enforcing access policies at cryptographic level
Searchable Encryption (SE, incl. PEKS/CP-ABSE)	Moderate: efficiency decreases with keyword complexity and policy size	Moderate: query processing slower than plaintext search	Can support compliance by enabling access-controlled and auditable queries on encrypted data

As summarized in Table 25, each privacy-preserving method presents distinct trade-offs in terms of scalability, latency, and regulatory compliance. ZKPs offer strong compliance benefits by enabling selective disclosure and consent enforcement, but their relatively high proof generation cost makes them less practical for latency-sensitive healthcare environments. HE ensures privacy-preserving analytics by allowing computation on encrypted data, yet the high computational overhead hinders scalability and real-time applicability. DP, in contrast, scales efficiently and introduces minimal latency, but the trade-off between privacy and data utility makes it better suited for population-level studies rather than individual-level healthcare applications.

PRE and CP-ABE provide flexible and fine-grained access control mechanisms, aligning well with compliance requirements such as GDPR and HIPAA. PRE achieves efficient data transfer and delegation but relies on trusted proxy operations. CP-ABE, while cryptographically enforcing complex access policies, introduces computational overhead in decryption and key management, which may limit adoption in resource-constrained environments.

#### Roadmap for the implementation of privacy-preserving blockchain solutions in healthcare

**Fig. 9:**
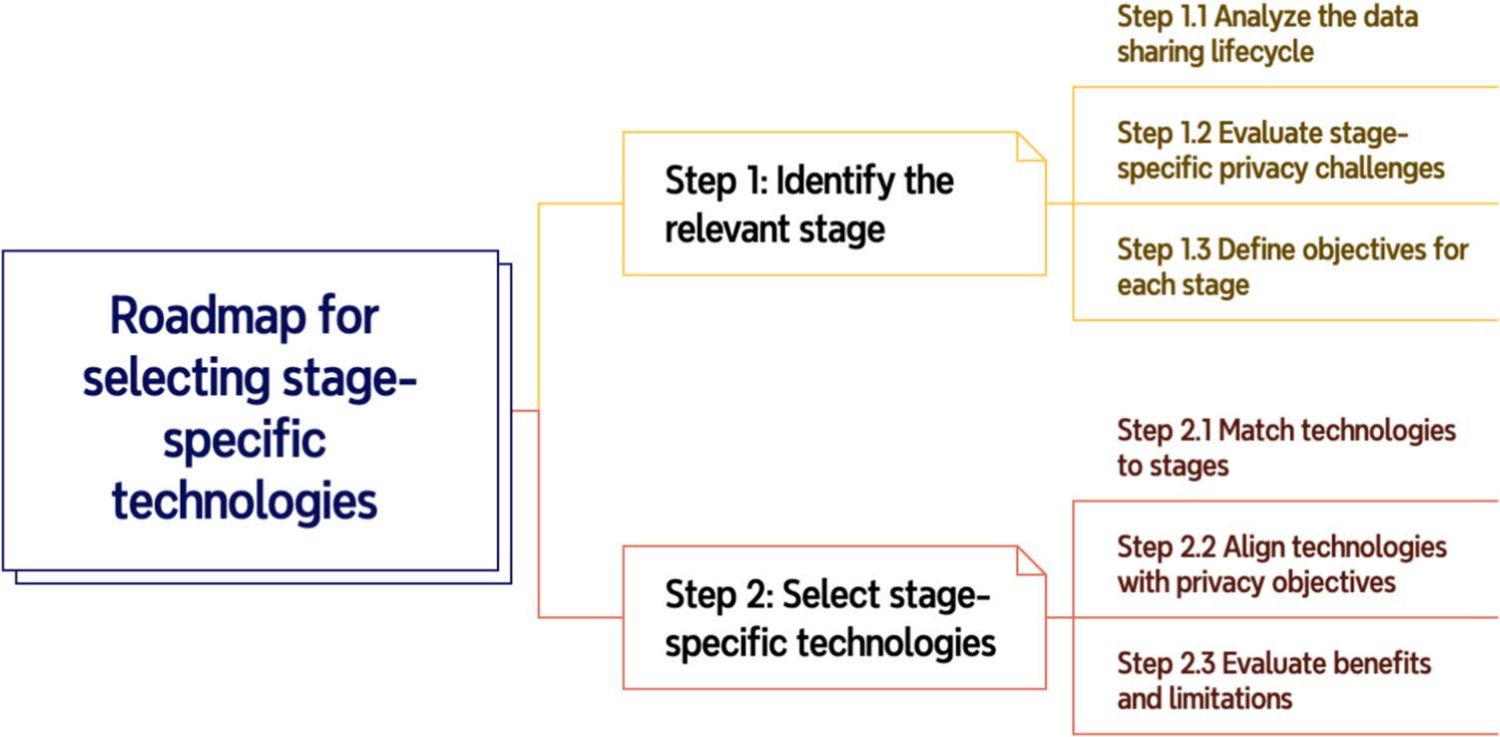
Roadmap for selecting privacy-preserving technologies in blockchain-based solutions healthcare data sharing

The identification of privacy-preserving methodologies across various stages of the data-sharing process fills the gap in the existing literature and provides a roadmap for selecting privacy-preserving blockchain solutions in healthcare, as shown in Fig 9. The selection of privacy-preserving technologies in blockchain-based healthcare data sharing must prioritize the protection of sensitive data while ensuring system functionality. This roadmap provides actionable guidelines centered on privacy requirements for each stage of the data-sharing process.

**Step 1: Identify the Relevant Data-Sharing Stage**

The roadmap for implementing privacy-preserving blockchain solutions in healthcare begins with identifying the relevant stage in the data-sharing process. This ensures that the technologies selected address the specific privacy challenges and requirements of each stage:

1. Analyze the Data Sharing Lifecycle: 1) Break down the data-sharing process into its five primary stages: data storage, search, verification, authentication and authorization, and transfer. 2) Determine the specific stage(s) where privacy concerns are most critical based on the nature of the data and the workflow requirements.
2. Evaluate Stage-Specific Privacy Challenges: 1) Data Storage: Assess risks related to secure storage, scalability, and compliance with regulatory standards. 2) Data Search: Identify challenges in enabling efficient and secure search functionality on encrypted data. 3) Verification: Focus on ensuring data authenticity, integrity, and accuracy without compromising sensitive information. 4) Authentication and Authorization: Analyze the need for robust access control and consent management mechanisms to safeguard sensitive data. 5) Data Transfer: Address potential vulnerabilities in transmitting sensitive data between entities while maintaining privacy and integrity.
3. Define Objectives for Each Stage: Specify the desired outcomes for privacy, security, and functionality at the identified stage(s) of concern.

**Step 2: Select Stage-Specific Technologies**

Once the relevant stage(s) of the data-sharing process have been identified, the next step involves selecting appropriate privacy-preserving technologies tailored to address the unique challenges and requirements of each stage.

1. Match Technologies to Stages: Leverage the insights from Sections 4.2.1 to 4.2.5 to identify technologies that align with the privacy and functional needs of the identified stage(s).
2. Align Technologies with Privacy Objectives: Ensure that the selected technologies address the privacy concerns identified, such as preventing unauthorized access, maintaining data integrity, or enabling anonymous sharing.
3. Evaluate Benefits and Limitations: Assess each technology’s strengths and potential drawbacks, as discussed in Sections 4.2.1 through 4.2.5.

**Fig. 10:**
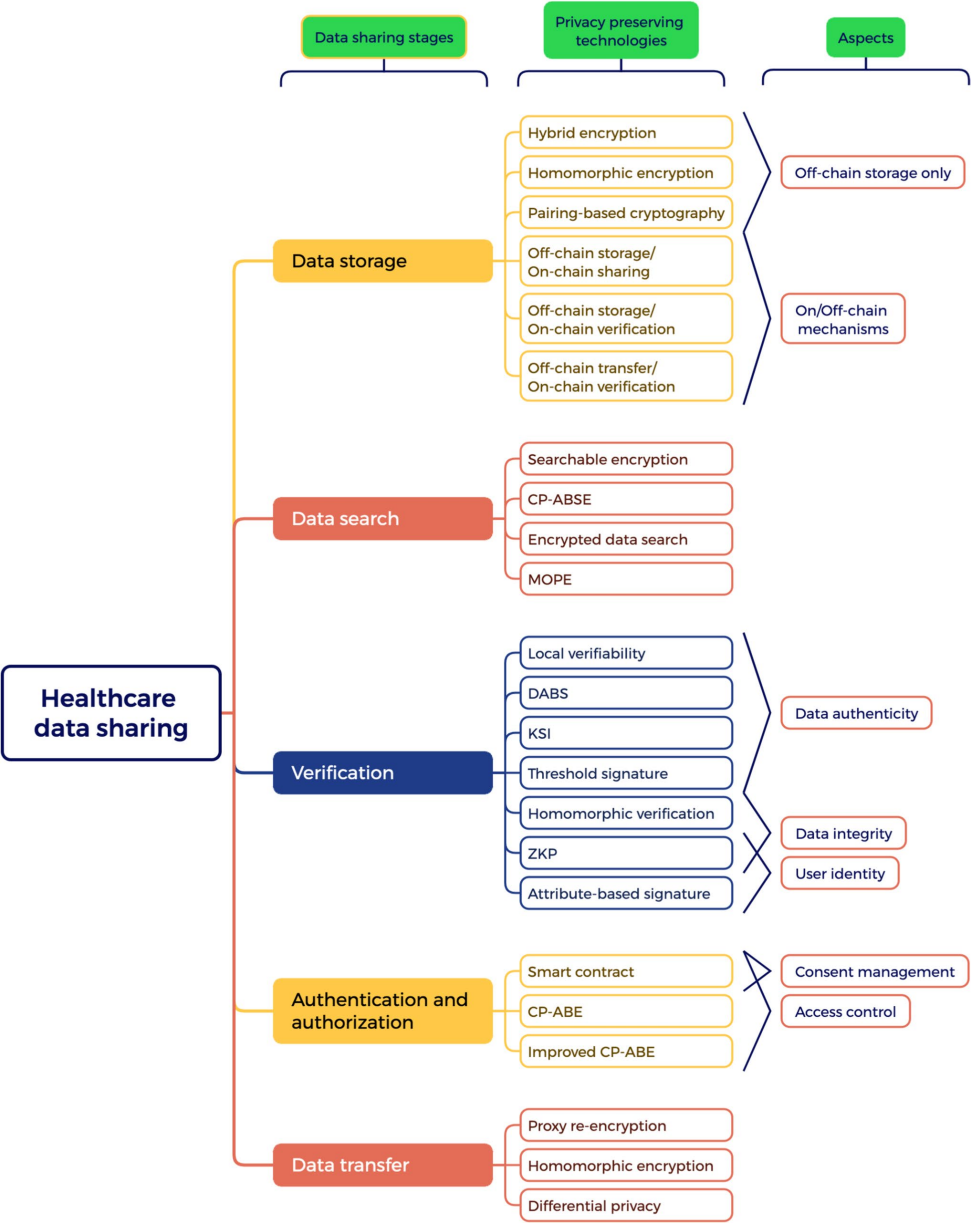
Overview for the implementation of privacy-preserving blockchain solutions in healthcare

To provide a clear overview of the privacy-preserving techniques and enhance the selection of privacy-preserving technologies, Fig. 10 summarizes the data sharing stages, associated technologies, and their respective application aspects. In this figure, the “data sharing stages” column represents the stages involved in healthcare data sharing, the “Privacy preserving technologies” column lists the technologies associated with each stage, and the “Aspects” column provides summaries of the scenarios where these technologies are applicable.

### Technology readiness and regulatory compliance in blockchain-based healthcare solutions

#### Technology readiness levels

This section evaluates the maturity of blockchain-based healthcare solutions using TRLs in Table 7, providing insights into the gaps and challenges that prevent broader adoption.

The majority of reviewed solutions are at TRL3, with only 37 papers reaching TRL4 and just one at TRL5. The predominance of studies at TRL3 signifies that most research remains at the concept validation stage, where ideas have only undergone limited testing in controlled laboratory environments. This predominance underscores a significant implementation gap, where researchers face challenges securing access to clinical data for realistic testing. Furthermore, insufficient collaboration between academia and healthcare institutions restricts the ability to validate solutions in practical settings.

**Fig. 11:**
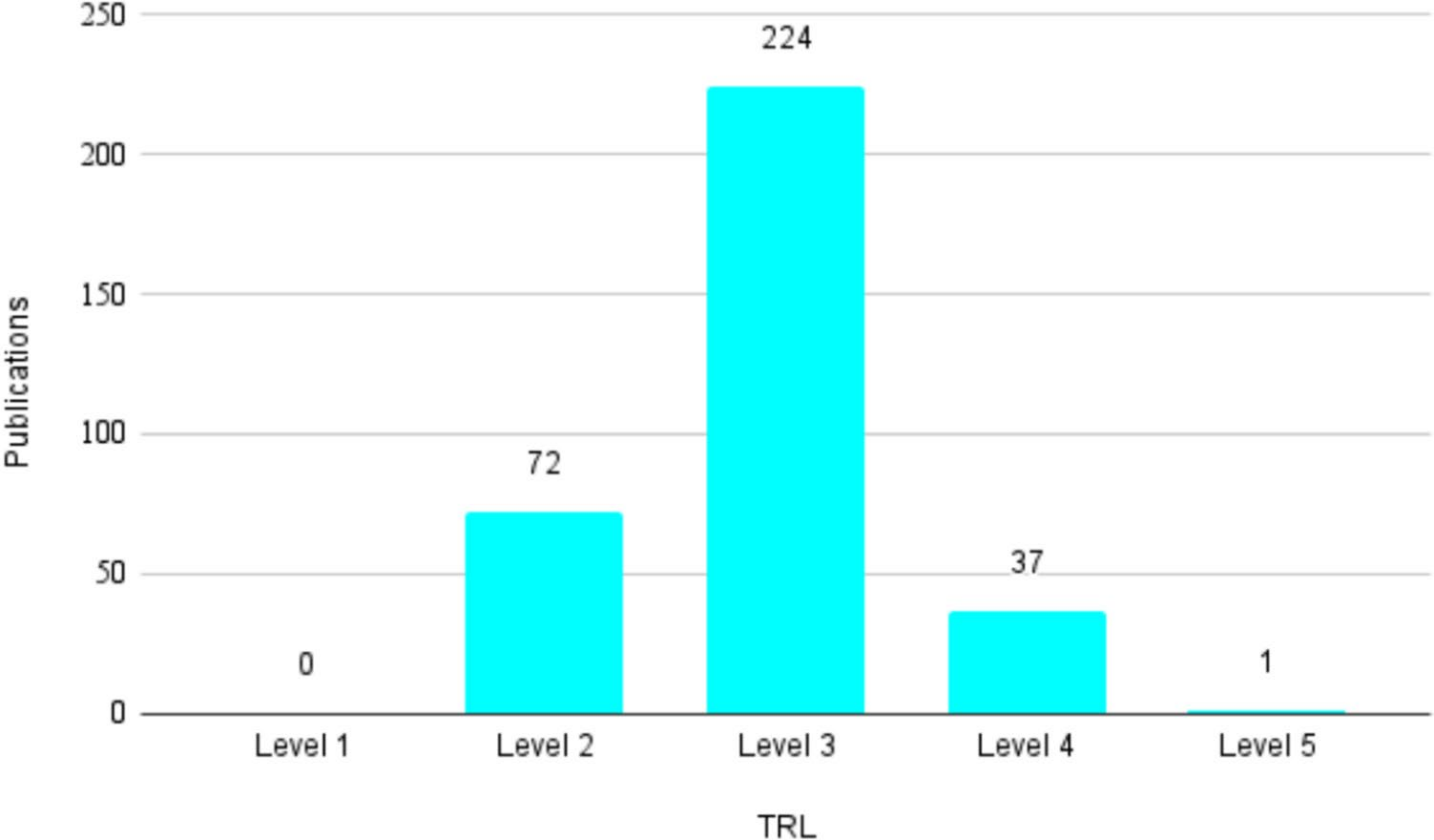
Technology Readiness Levels

Advancing from concept validation (TRL3) to real-world implementation (TRL4 and TRL5) remains a significant burden for academic blockchain solutions in healthcare. Although 37 solutions have reached TRL4, involving clinical data validation in laboratory settings, only one study achieved TRL5, which includes clinical trials or practical implementation, shown in Fig. 11.

The absence of blockchain-based healthcare solutions at TRL6 and beyond highlights a significant challenge in transitioning from academic prototypes to practical, scalable applications. While this study exclusively focused on academic initiatives, which may partly explain this absence, other contributing factors also play a role, such as lack of industry partnerships, technical scalability issues, and regulatory compliances.

#### Regulatory compliance

In the reviewed papers, eight papers conducted regulatory compliance assessments. Table 26 summarizes how these studies address healthcare privacy regulations. The most commonly cited frameworks are HIPAA and GDPR, with one solution also referencing APPs and another citing HITECH.

**Table 26:** Summary of blockchain-based healthcare data-sharing solutions and regulatory compliance

Reference	Regulation(s)	Evaluation of Regulatory Compliance	Compliance Challenges
[27]	HIPAA, HITECH	1. Implements HIPAA Privacy Rule via smart-contract-based access control 2. Aligns with HIPAA Security Rule with identity verification 3. HITECH: focuses on interoperability by enabling nationwide electronic exchange	1. Lack of full anonymity; potential indirect inference attacks 2. Data immutability vs. HIPAA “revocation of access” (Right to be Forgotten)
[28]	GDPR	1. Allow Right to be Forgotten with smart contract-based consent 2. Allow Purpose-Based Access Control by implementing consent code in smart contract	Practical enforcement of Right to be Forgotten while maintaining blockchain integrity
[186]	GDPR	1. Patient-centric access control using CP-ABE 2. Fine-grained access policies enforced via on-chain approval	Blockchain’s immutability conflicts with data-erasure requirement
[24]	HIPAA	1. Sensitive PHI stored off-chain for HIPAA compliance 2. Smart contracts enforce access rules; on-chain logs provide accountability	Ensuring secure off-chain data storage
[184]	HIPAA	Full HIPAA compliance	Consistent HIPAA compliance across multiple stakeholders
[185]	GDPR	1. Right to be Forgotten: Data stored in IPFS can be removed at user request 2. Authentication and access control via smart contracts	1. Blockchain’s immutability conflicts with data-erasure requirement2. Ensuring effective data access control for multiple parties
[25]	GDPR	Full GDPR compliance	Blockchain’s transparency ensures data integrity but can conflict with privacy regulations
[26]	Australian Privacy Principles(APPs), GDPR	1. Aligns with APPs (open/transparent management, anonymity, data minimization) 2. Aligns with GDPR core principles (lawfulness, fairness, storage limitation)	1. Blockchain’s immutability conflicts with data-erasure requirement 2. Need for robust key management and encryption

##### Prevalent regulations focus

 

- **HIPAA/HITECH**: Multiple solutions [24, 27, 184] center on HIPAA compliance, largely focusing on the Privacy Rule (protecting personal health information) and the Security Rule (defining administrative and technical safeguards). HITECH often appears alongside HIPAA to address interoperability concerns [27].
- **GDPR**: Primarily emphasized in solutions that aim to enable patient data rights [25, 28, 185, 186]. GDPR discussions often spotlight data minimization, lawful processing, and user consent.
- **APPs**: Only one solution [26] references the Australian Privacy Principles, highlighting the importance of transparent data practices and anonymization within Australian healthcare contexts

##### Claimed compliance

Two studies explicitly claim “full compliance” [25, 184]. The authors in Sarier [25] present a Privacy-Preserving Biometric Authentication (PPBA) protocol designed to be fully compliant with GDPR. To achieve this, they implement an off-chain storage mechanism using a Merkle tree, preventing the direct exposure of sensitive biometric data on the blockchain and ensuring users have control over their information. The system allows users to revoke their biometric templates by replacing them with randomized entries, thereby supporting the “Right to Erasure” principle. Furthermore, the authentication mechanism relies on ZKPs and Monero’s privacy-enhancing blockchain infrastructure to protect identity privacy, ensuring that no biometric data can be linked to a specific user. Additionally, the system provides an auditable framework, recording authentication steps as blockchain transactions, allowing for regulatory oversight and compliance verification. To ensure regulatory compliance, the authors of HealthChain [184] aligned their framework with HIPAA by integrating multiple security and privacy measures. The system enforces authorization and access control through smart contracts, allowing verifiable user permissions and secure data sharing mechanisms. PRE, hashing key pairs and digital signatures are employed to safeguard data integrity and security, ensuring authentication and protection against unauthorized access. Furthermore, auditability is maintained through log blocks, providing a transparent and verifiable trail of modifications and access. In terms of transmission security, the framework utilizes re-encryption layers and access verification mechanisms to prevent data breaches during exchanges

In contrast, other studies provide partial evaluations, mapping their techniques to specific regulatory articles [24, 26–28, 185, 186]. As summarized in Table 26, which presents the Evaluation of Regulatory Compliance, we outline the regulatory articles addressed by these techniques, offering a clear overview of how these solutions adhere to relevant regulations. For example, the solution in Bai et al. [185] supports data deletion from IPFS through a proof of ownership. The data owner can request deletion by providing a hashed version of the file and a content-dependent key, ensuring the file is permanently removed. The blockchain does not store personal data, only metadata or hashes, preventing the storage of sensitive information. In this way, the solution complies with the “Right to Be Forgotten” in GDPR.

##### Recurring compliance challenges

Nearly all GDPR-oriented solutions [26, 28, 185, 186] cite the friction between the blockchain and the “Right to Be Forgotten (RTBF).” The RTBF grants individuals the right to request the erasure of their data, ensuring that information is removed when it is no longer necessary or when consent is withdrawn. However, blockchain operates as an append-only ledger, meaning that once data is recorded, it cannot be deleted or modified. This fundamental property ensures data integrity and prevents tampering but creates legal and technical challenges in scenarios where data erasure is required.

Proposed workarounds in these solutions [26, 28, 185, 186] mainly include deleting or re-encrypting off-chain data and revoking access in smart contracts, although the data pointers on-chain remain theoretically recoverable. In this way, privacy is preserved by limiting on-chain exposure and enabling practical data “erasure.” Rather than embedding personal data in the blockchain itself, only references are stored on-chain, so once the off-chain data is deleted or its keys are invalidated, unauthorized access becomes infeasible. This approach aligns with the “Right to Be Forgotten” by effectively removing an individual’s data from use while retaining the blockchain’s immutability. Although absolute irrecoverability cannot be guaranteed–since on-chain pointers persist–this method offers a balanced compromise that upholds privacy protections without undermining the fundamental immutability of the blockchain ledger.

## Discussion

The analysis of data collected from the selected papers has provided insights into the research interest trend, blockchain technology details, privacy-preserving methods, and TRLs of preserving privacy while sharing healthcare data based on blockchain technology. In this section, we present a discussion by answering the research questions proposed before.

### Implementation patterns and framework for blockchain-based healthcare data sharing

Blockchain-based healthcare data sharing solutions can vary significantly, depending on the specific objectives for privacy, scalability, and interoperability. This section explores **RQ1: How is blockchain technology applied to healthcare data sharing?**

As illustrated in Section 4.1, this study provides insights into how blockchain technology is applied to healthcare data sharing by analyzing three factors: blockchain types, consensus protocols, and blockchain implementations. Additionally, this study identifies two distinct application patterns: the consortium blockchain pattern and the public permissionless blockchain pattern. These factors and patterns reflect the diverse requirements in healthcare data sharing, from privacy and controlled access to openness and decentralization.

The identification of these two patterns forms the basis for a framework to guide the design of privacy-preserving blockchain solutions in healthcare, as shown in Section 4.1.4. This framework offers developers and researchers actionable guidance for constructing blockchain systems tailored to healthcare data sharing. By aligning system design with specific goals–whether prioritizing privacy or openness–it enables stakeholders to select the pattern that best meets their needs, effectively balancing competing priorities within the healthcare sector.

Although the framework provides valuable theoretical insights and highlights trade-offs in system design, it has yet to be empirically validated. Future research should focus on implementing and testing this framework in real-world healthcare environments to evaluate its practicality and effectiveness. Through pilot studies and experiments, researchers can refine the identified patterns, uncover additional trade-offs, and optimize the framework to better address the complexities of healthcare applications. Such efforts would ensure that the framework aligns with the specific needs of stakeholders, offering a balance between scalability, privacy, and efficiency.

Despite these advancements, several challenges remain. Scalability issues, particularly in public blockchains like Ethereum, hinder their use in high-volume scenarios. Moreover, the trade-off between privacy and transparency introduces data re-identification risks, further complicating adoption. Finally, as shown in Section 4.3, most blockchain implementations in healthcare are limited to the concept validation stage, lacking real-world application and scalability.

To overcome these challenges, future research should prioritize the development of scalable, privacy-compliant blockchain mechanisms. Furthermore, fostering real-world implementations and testing these solutions in practical settings will be critical for advancing the maturity of blockchain-based healthcare systems. Addressing these barriers will pave the way for broader adoption of blockchain technology in healthcare, enhancing data sharing while maintaining privacy protections.

### Privacy-preserving technologies and implementation roadmap in blockchain-based healthcare data sharing

A key focus of this review is to determine which privacy-preserving technologies are most commonly integrated into blockchain-based healthcare data sharing solutions (**RQ2: Which privacy-preserving technologies are used in blockchain-based health data sharing solutions?**). By examining how such technologies apply to each stage of data sharing–from storage through transfer–this section proposes an actionable roadmap to ensure both confidentiality and system integrity, as shown in Section 4.2.7.

The identification of privacy-preserving methodologies across various stages of the data-sharing process fills the gap in the existing literature. By illustrating specific technologies suited to distinct stages, it emphasizes the importance of addressing privacy continuously, ensuring data protection from storage to transfer. The exploration of methodologies such as the on/off-chain mechanism, encryption-based searches, and smart contracts demonstrates how blockchain can be designed to balance privacy, performance, and scalability. This balance is important in healthcare environments, where regulations such as HIPAA and GDPR must be strictly followed.

Building on these identified technologies, this study offers a roadmap for selecting privacy-preserving technologies in healthcare, as presented in Fig. 9. The roadmap serves as actionable guidance for developers and researchers, providing guidance on choosing privacy preserving technologies while supporting the efficiency, scalability, and interoperability demanded by modern healthcare systems. This approach is essential for the integration of privacy preserving technologies in healthcare, supporting the secure and ethical handling of sensitive information. This guidance can aid stakeholders in adopting privacy preserving technologies that safeguard patient data, making blockchain-based privacy-preserving solutions more feasible and impactful across the healthcare sector.

Nonetheless, several challenges remain. As shown in Sections 4.2.6 and 4.2.7, no single technique can simultaneously optimize scalability, latency, and compliance. Hybrid approaches–such as combining PRE for efficient data transfer with ZKPs for consent verification, or integrating DP with HE for privacy-preserving analytics–offer a more balanced pathway toward deployment. In practice, this requires system designers to select and combine methods according to the needs of specific healthcare scenarios, such as real-time patient monitoring, population-level research, or cross-institutional data exchange.

At the same time, research remains uneven across different stages of data sharing. In particular, privacy-preserving search and verification have received comparatively little attention, exposing gaps in comprehensive adoption. Addressing these gaps will require tackling computational constraints, establishing standardized privacy frameworks, and expanding research efforts across all data-sharing stages. Together, these directions are essential for advancing blockchain-based privacy-preserving solutions toward broader adoption in healthcare.

### Technology readiness and regulatory compliance in blockchain-based healthcare data sharing

This section examines both the current maturity of privacy-preserving blockchain solutions (TRLs) and their alignment with regulations. Taken together, these factors address the question of how established blockchain-based healthcare solutions truly are in practice (**RQ3: To what extent privacy-preserving solutions based on blockchain are established in healthcare?**).

#### Technology readiness levels of blockchain-based privacy-preserving solutions

The findings of this review reveal that privacy-preserving solutions in blockchain-based healthcare data sharing have gained significant attention and made notable progress over the years, as shown in Fig. 4. The increasing number of publications per year demonstrates a steady upward trend, with notable growth in recent years. This indicates a growing recognition among researchers and practitioners of blockchain technology’s potential to address privacy concerns in healthcare data sharing. However, the TRL evaluation, presented in Section 4.3.1, shows that the majority of blockchain-based privacy-preserving solutions in healthcare are at the early stages of development, with most solutions assessed at TRL 3. Only 29 studies progressed to TRL4, and just one study achieved TRL5. No solutions have reached higher TRLs (TRL6 and above), which represent full-scale deployment in real-world clinical environments.

The predominance of studies at TRL3 highlights a strong emphasis on conceptual validation in laboratory settings. While this demonstrates the feasibility of proposed solutions, it also underscores a lack of pilot studies and clinical trials necessary for real-world adoption. Furthermore, the absence of solutions at TRL6 and beyond reflects the challenges in transitioning from prototypes to market-ready applications. This gap indicates a need for greater focus on implementation, interoperability, and user engagement. Most studies concentrate on technical innovations, often neglecting the integration of these solutions into existing healthcare workflows. This oversight may hinder practical deployment and user acceptance.

It is important to note that a limitation in evaluating TRL from academic papers: the focus on academic research overlooks potential advancements in industry products, which may not be published in academic literature. Many industry-led solutions might already operate at higher TRLs, particularly through proprietary systems, private collaborations, or non-academic platforms. This limitation emphasizes the need for future reviews to incorporate broader datasets, including industry reports and gray literature, to provide a more comprehensive view of the state of development.

To bridge the gap and advance the maturity of blockchain-based privacy-preserving solutions in healthcare, future research must prioritize the transition from conceptual models to pilot implementations. This requires collaboration with healthcare institutions to secure access to clinical environments and datasets. Moreover, solutions should incorporate mechanisms to address privacy regulations, such as off-chain storage for sensitive data or privacy-preserving updates to immutable records. Fostering partnerships between researchers and industry stakeholders can bridge the gap between theoretical solutions and practical applications, facilitating resource sharing, real-world testing, and eventual commercialization. Additionally, addressing technical challenges–such as scalability, cost-efficiency, and system integration–will be essential for advancing these solutions to higher TRLs.

#### Regulatory compliance: Current status and challenges

Beyond technological feasibility and maturity levels, regulatory compliance is significant in determining whether blockchain-based healthcare solutions can transition from prototypes to fully operational, real-world products. As highlighted in Table 26, in the studies with regulatory assessment, most of them claim compliance or partial compliance with regulations such as HIPAA, GDPR, or APPs. However, these claims often remain at a conceptual or prototype stage, mirroring the TRL findings where the majority of solutions are assessed at TRL3. In other words, while researchers are cognizant of the legal frameworks, practical demonstration of compliance within real clinical settings is still limited.

Although many solutions propose off-chain storage, key-revocation mechanisms, or consent management smart contracts to address privacy requirements (e.g., GDPR’s Right to Erasure), they are tested only in laboratory environments. Besides, True regulatory compliance typically requires third-party audits or certifications (e.g., HITRUST for HIPAA). However, most studies at TRL3 only assert theoretical compliance without external validation or pilot programs in active healthcare facilities.

Moving from conceptual compliance to verified, real-world compliance is essential for solutions to reach TRL6+. Higher TRLs require full operational testing, including alignment with existing clinical processes and secure data exchange. For GDPR-related solutions, this might involve real patient consent procedures, deletion requests, and multi-jurisdiction data sharing. Besides, Implementing robust privacy-preserving mechanisms, such as ZKPs or off-chain data encryption, can introduce computational overhead. Future research must demonstrate that these privacy-preserving features remain efficient and scalable when deployed in large health networks.

Future works should collaborate with healthcare stakeholders because it is crucial for conducting pilot implementations under real governance, legal, and workflow constraints. Formal audits or certifications can confirm whether these blockchain-based solutions fulfill requirements, thereby enhancing trust among practitioners and regulators. Besides, academic studies typically emphasize conceptual or prototype phases (TRL1–4). Industry-led projects or private consortiums may already operate at higher TRLs but often publish little in peer-reviewed venues. Future reviews and collaborations should expand beyond academic databases to capture industry white papers, patents, or technical reports that might reveal more advanced implementations.

### Privacy vs. security: Interplay and challenges

While privacy preservation is critical in blockchain-based healthcare data sharing, it cannot be fully isolated from security considerations. The unique characteristics of blockchain systems, like transparency, immutability and decentralization, introduce a range of security threats, including Denial-of-Service (DoS) attacks, Sybil attacks, and smart contract vulnerabilities, which may undermine privacy guarantees if not adequately addressed.

#### Security threats in blockchain healthcare systems

PoW consensus mechanisms, widely used in public blockchains like Bitcoin and Ethereum (pre-Merge), are susceptible to DoS attacks due to their reliance on computational resources. Attackers can flood the network or exploit high energy demands to disrupt normal operations. Furthermore, Sybil attacks–where a single adversary controls multiple nodes–pose a risk to network integrity. While PoW mitigates Sybil attacks through economic cost, other consensus protocols such as Proof-of-Stake (PoS) and Practical Byzantine Fault Tolerance (PBFT) implement alternative defenses like stake-based validation or message-based agreement among trusted validators. However, PBFT’s reliance on a fixed validator set in permissioned blockchains introduces centralization risks that may reduce resilience against coordinated attacks.

Smart contracts also bring new security challenges. Exploits such as reentrancy attacks and integer overflows have led to significant losses in blockchain ecosystems. In healthcare contexts, compromised smart contracts could result in unauthorized access to sensitive patient data or disruption of consent management systems. As healthcare solutions increasingly rely on complex smart contracts for access control and data sharing, ensuring their correctness and security becomes essential.

#### Interplay between privacy and security

Privacy-preserving techniques, such as ZKPs and ring signatures, often introduce additional computational and storage overhead. This may inadvertently increase susceptibility to DoS attacks, particularly in resource-constrained healthcare environments. Moreover, some security measures designed to prevent Sybil attacks or ensure regulatory compliance (e.g., mandatory KYC procedures) may conflict with privacy goals by reducing user anonymity.

Conversely, strong security mechanisms can enhance privacy. For instance, robust Sybil-resistance prevents adversaries from aggregating pseudonymous transactions to deanonymize users. Similarly, secure smart contract design reduces the risk of privacy breaches due to logic flaws or exploits.

#### Challenges and future directions

Our systematic review indicates that while privacy preservation is the primary focus of most blockchain-based healthcare data sharing solutions, explicit considerations of security–such as resistance to Denial-of-Service (DoS) attacks, Sybil-resistance, and smart contract vulnerabilities–are less frequently discussed. However, as healthcare systems increasingly interface with public blockchains or hybrid architectures, the need to balance privacy and security becomes more pronounced. Future work could benefit from a more holistic design approach, where privacy-preserving techniques (e.g., zero-knowledge proofs) are evaluated alongside security mechanisms to ensure resilience against both privacy breaches and systemic attacks.

### Methodological considerations and limitations

While we discussed specific limitations in relation to each research question, several overarching limitations of this review should be noted. First, the search strategy was restricted to major databases. Grey literature such as whitepapers, technical reports, or non–peer-reviewed preprints was not included, which may have led to the omission of some emerging blockchain-based approaches that are disseminated outside traditional academic venues. Second, although Technology Readiness Levels (TRLs) were used to evaluate the maturity of the selected solutions, the accuracy of TRL assignment depends on the level of detail reported in individual studies, which varied considerably. These limitations should be considered when interpreting the findings of this review.

## Conclusion

This paper presents a systematic review of the current state of privacy-preserving solutions in blockchain-based healthcare data sharing. Through an analysis of 334 papers, we have identified trends in applying blockchain, patterns in designing blockchain-based solutions, and privacy-preserving methodologies used in these solutions. We have also evaluated the maturity and regulatory compliance of these solutions. Furthermore, we have developed a framework for blockchain-based healthcare data-sharing system design and a roadmap for the selection of privacy-preserving technologies in healthcare.

Our findings show that blockchain is being widely applied in healthcare data sharing, with consortium blockchains emerging as the dominant choice due to their ability to control access and ensure higher privacy. Public permissionless blockchains, such as Ethereum, are also gaining traction for their decentralization and openness, though they come with performance trade-offs. These insights into blockchain types, consensus protocols, and implementations provide a framework for developers and researchers aiming to balance privacy and openness in healthcare applications.

Regarding privacy preservation, the review highlighted various methodologies employed at different stages of the data-sharing process. Furthermore, this review developed a roadmap for integrating privacy-preserving technologies in healthcare for developers and researchers, aiding them in adopting proper technologies that safeguard patient data, making blockchain-based privacy-preserving solutions more feasible and impactful across the healthcare sector.

Despite the advancements, our evaluation of TRLs reveals that most solutions are still at the concept validation stage (TRL3). While there has been significant progress in laboratory settings, few solutions have been tested in real-world healthcare environments (TRL4 or TRL5). This highlights the need for further collaboration between academic researchers and healthcare institutions to advance blockchain-based privacy-preserving solutions towards clinical implementation and broader market adoption.

In conclusion, blockchain holds considerable potential to address privacy concerns in healthcare data sharing. However, achieving full-scale adoption will require overcoming implementation challenges, particularly around scalability, interoperability, and regulatory compliance. Future research should focus on bridging the gap between experimental solutions and practical applications while fostering stronger partnerships to facilitate clinical validation and real-world testing. Our systematic review serves as a foundation for ongoing innovation in privacy-preserving blockchain technologies and their application in the healthcare sector.

## Data Availability

Data is provided within the manuscript.

## Appendix A: Terms and filters used while searching from data sources

**Table 27:** Terms and filters used while searching from data sources

Data sources	Searching terms	Filters
IEEE Xplore	(“All Metadata”:blockchain) AND (“All Metadata”:privacy) AND (“All Metadata”:healthcare) AND (“All Metadata”:sharing)	Conferences; Journals; 2008-2023
SpringerLink	blockchain AND privacy AND healthcare AND sharing AND NOT (survey AND review)	2008-2023
ScienceDirect	blockchain AND privacy AND healthcare AND sharing	Year: 2008-2023; Title, Abstract, Key words: NOT survey NOT review
Web of Science	blockchain (All Fields) and healthcare (All Fields) and privacy (All Fields) and sharing (All Fields)	Document Type: Article or Proceeding Paper; Timespan: 2008-01-01 to 2023-12-31
ACM Digital Library	[All: blockchain] AND [All: healthcare] AND [All: privacy] AND [All: sharing] AND NOT [Title: review] AND NOT [Title: survey] AND NOT [Keywords: review] AND NOT [Keywords: survey] AND NOT [Abstract: review] AND NOT [Abstract: survey] AND [Publication Date: (01/01/2008 TO 12/31/2024)]	Research Article; Proceedings or Journals

## Appendix B: Data sources for this systematic review

**Table 28:** Data Sources for This Systematic Review

Paper	Regulatory compliance	Technologies	Data sharing stage	Blockchain Type	Blockchain Implementation	Consensus Protocol	TRL
[187]	No	purpose-centric access control	AA	Not Discussed	Not Discussed	Not Discussed	2
[147]	No	an original decentralized attribute-based signature/on-off chain storage	verification; Storage	Consortium	Custom-built	PBFT	3
[124]	No	smart contract	AA	Public	Ethereum	PoW	2
[27]	HIPAA; HITECH	proxy re-encryption/smart contract	Transfer; AA	Public	Ethereum	PoW	2
[128]	No	on-off chain storage	Storage	Hybrid	Custom-built	PoW	3
[161]	No	smart contract	AA	Consortium	ethereum	PoA	2
[188]	No	Tor protocol	Transfer	Private	Ethereum and Hyperledger	PoW	4
[153]	No	content extraction signature/smart contract	AA	Consortium	Not Discussed	DPoS	2
[189]	No	public key/private key encryption	Storage	Public	Custom-built	Not Discussed	3
[152]	No	on-off chain storage/smart contract	Storage; AA	Private	Ethereum	PoW	3
[145]	No	Attribute-based signature	verification	Not Discussed	Not Discussed	Not Discussed	2
[190]	No	combined attribute-based/identity-based encryption and signature (C-AB/IB-ES).	AA	Consortium	Not Discussed	Not Discussed	2
[183]	No	differential privacy	Transfer	Not Discussed	Bitcoin	PoW	4
[191]	No	BBL-CL-PKC	AA	Consortium	Not Discussed	Not Discussed	2
[169]	No	smart contract	AA	Not Discussed	Not Discussed	Not Discussed	2
[93]	No	original distributed storage/proxy re-encryption	Storage; Transfer	Hybrid	Custom-built	DPoS-Quorum	5
[87]	No	smart contract	AA	Consortium	Hyperledger Fabric	PBFT	3
[192]	No	sibling intractable function families	AA	Public	Hyperledger Fabric	PBFT	3
[108]	No	proxy re-encryption	Transfer	Consortium	hyperledger fabric	PBFT	3
[193]	No	zkp/proxy re-encryption	verification; Transfer	Consortium	hyperledger fabric	PBFT	3
[28]	GDPR	smart contract	AA	Public	Ethereum	PoW	4
[136]	No	PRE/PEKS/PUF	Transfer; search; Transfer	Hybrid	Not Discussed	Not Discussed	2
[160]	No	smart contract	AA	Private	ethereum	PoW	3
[194]	No	blind signature	AA	Not Discussed	Not Discussed	Not Discussed	2
[125]	No	data division/smart contract	Storage; AA	Public	Ethereum	PoW	3
[137]	No	deniably authenticated encryption technology	search	Not Discussed	Not Discussed	PBFT	2
[195]	No	hybrid signature/Merkle tree optimization model	AA	Not Discussed	Not Discussed	raft	4
[154]	No	smart contract/on-off chain storage	AA/Storage	Hybrid	Ethereum	PoW/PoA	3
[70]	No	PEKS/proxy re-encryption	search; Transfer	Hybrid	Not Discussed	Not Discussed	2
[86]	No	smart contract	AA	Public	Ethereum	PoW	3
[186]	GDPR	Attribute-based encryption	AA	Hybrid	Custom-built	PBFT	3
[158]	No	smart contract	AA	Consortium	Hyperledger Fabric	PBFT	3
[113]	No	Attribute-Based Encryption	AA	Public	Ethereum	PoW	3
[148]	No	keyless signature infrastructure	verification	Not Discussed	Not Discussed	Not Discussed	2
[196]	No	identity authentication chain	AA	Hybrid	Not Discussed	PoW; PBFT	2
[131]	No	double encryption	Storage	Private	Ethereum	PoA	3
[71]	No	searchable encryption/smart contract	search; AA	Private	Ethereum	PoW	4
[197]	No	trust computing	AA	Public	Not Discussed	Not Discussed	2
[198]	No	onion structure	Transfer	Private	Ethereum	PoW	3
[159]	No	federated learning	Transfer	Public	Ethereum	PoW	4
[199]	No	customed access policies	AA	Consortium	Custom-built	PoA	3
[106]	No	Quantum blind signature	AA	Consortium	hyperledger Fabric	PBFT	3
[200]	No	differential privacy	Transfer	Not Discussed	Not Discussed	Not Discussed	2
[201]	No	Merkle-Hellman knapsack cryptosystem	Transfer	Not Discussed	Not Discussed	Not Discussed	2
[202]	No	on-off chain storage	Storage	Consortium	Hyperledger Fabric	PBFT	3
[203]	No	role-based AA	AA	Public	Custom-built	self-design	3
[114]	No	decentralized machine learning	Transfer	Public	Ethereum	PoW	4
[104]	No	consortium blockchain & IPFS	Storage	Consortium	Hyperledger Fabric	PBFT	3
[24]	HIPAA	on-off chain storage	Storage	Private	ethereum	PoW	2
[204]	No	H-PCS	AA	Consortium	hyperledger fabric	PBFT	4
[102]	No	identity-based broadcast group signcryption	AA	Consortium	Hyperledger Fabric	PBFT	2
[205]	No	steganography	Transfer	Not Discussed	Not Discussed	Not Discussed	2
[126]	No	smart contract	AA	Consortium	Hyperledger Fabric	PBFT	3
[206]	No	MPC	Storage	Public	Ethereum	PoW	3
[207]	No	proxy re-encryption	Transfer	Public	Bitcoin	BFT-SMaRt	3
[208]	No	on-off chain storage	Storage	Consortium	Not Discussed	PoS	2
[172]	No	improved CP-ABE	AA	Consortium	CITA	PBFT	3
[209]	No	differential privacy	Transfer	Not Discussed	Not Discussed	Not Discussed	3
[156]	No	smart contract	AA	Not Discussed	Not Discussed	Not Discussed	2
[210]	No	message sharing scheme	Transfer	Not Discussed	Not Discussed	Not Discussed	3
[211]	No	ring signature	AA	Private	Hyperledger Fabric	PBFT	3
[157]	No	smart contract	AA	Private	Ethereum	PoW-infer	3
[212]	No	on-off chain storage	Storage	Consortium	Hyperledger Fabric	PBFT	3
[213]	No	proxy re-encryption	Transfer	Consortium	Hyperledger Fabric	PBFT	2
[112]	No	on-off chain storage	Storage	Public	Ethereum	PoW	3
[214]	No	ring signature	AA	Not Discussed	Not Discussed	Not Discussed	2
[103]	No	smart contract	AA	Consortium	Hyperledger Fabric	PBFT	3
[215]	No	proxy re-encryption	Transfer	Public	Ethereum	PoW	3
[155]	No	smart contract	AA	Public	Ethereum	PoW	3
[79]	No	on-off chain storage	Storage	Consortium	Hyperledger Fabric	PBFT	4
[216]	No	certificateless aggregate signature	AA	Not Discussed	Not Discussed	Not Discussed	3
[110]	No	proxy re-encryption	Transfer	Consortium	Hyperledger Fabric	PBFT	3
[171]	No	smart contract	AA	Consortium	Hyperledger Fabric	PBFT	3
[184]	HIPAA	proxy re-encryption	Transfer	Consortium	Hyperledger Fabric	PBFT	3
[129]	No	on-off chain storage/Tor	Storage; Transfer	Public	Ethereum	PoA	3
[217]	No	Traceable Ring Signature	AA	Not Discussed	Not Discussed	Not Discussed	2
[218]	No	Masked authenticated messaging/PUF	Transfer; AA	Not Discussed	IOTA	Not Discussed	3
[219]	No	CP-ABE	AA	Consortium	Not Discussed	Not Discussed	3
[220]	No	fuzzy extractor	AA	Not Discussed	Not Discussed	Not Discussed	3
[221]	No	smart contract	AA	Consortium	Ethereum	PoW-infer	3
[163]	No	smart contract	AA	Not Discussed	Not Discussed	Not Discussed	3
[222]	No	role-based account model	AA	Not Discussed	original	Proof of Transactions	2
[204]	No	AccessRuleTable	AA	Not Discussed	Hyperledger Fabric	PBFT-infer	3
[223]	No	Secure Multi-party Computation/Threshold Cryptography	Transfer; AA	Private	Not Discussed	Not Discussed	3
[224]	No	homomorphic encryption	Transfer	Consortium	Hyperledger Fabric	PBFT-infer	3
[225]	No	proxy re-encryption	Transfer	Not Discussed	Ethereum	PoW-infer	3
[226]	No	differential privacy	Transfer	Consortium	Not Discussed	Not Discussed	2
[227]	No	homomorphic encryption	Transfer	Not Discussed	Not Discussed	Not Discussed	3
[228]	No	federated learning	Transfer	Not Discussed	Not Discussed	Not Discussed	3
[229]	No	Hyperledger fabric channel	Transfer	Private	Hyperledger Fabric	PBFT-infer	2
[151]	No	zkp	verification	Public	Ethereum/Hyperledger Sawtooth	PoW-infer	3
[165]	No	smart contract	AA	Public	Ethereum	PoW-infer	3
[230]	No	federated learning	Transfer	Not Discussed	Not Discussed	Not Discussed	2
[231]	No	differential privacy	Transfer	Not Discussed	Custom-built	Robust Proof-of-Stake	3
[232]	No	attribute-based encryption	AA	Not Discussed	Hyperledger Fabric	PBFT-infer	3
[233]	No	differential privacy	Transfer	Not Discussed	Ethereum	PoW-infer	4
[234]	No	CP-ABE	AA	Consortium	Hyperledger Fabric	PBFT-infer	3
[235]	No	smart contract	AA	Consortium	Hyperledger Fabric	PBFT-infer	3
[236]	No	proxy re-encryption	Transfer	Not Discussed	Ethereum	PoW-infer	3
[237]	No	attribute-based encryption	AA	Consortium	Hyperledger Fabric	PBFT-infer	3
[238]	No	proxy re-encryption	Transfer	Not Discussed	CosmWasm; Hyperledger Fabric; Ethereum	Tendermint; Istanbul Byzantine Fault Tolerance 2; PoS	3
[239]	No	new key agreement and authentication protocol	AA	Not Discussed	Not Discussed	Not Discussed	3
[169]	No	ring signature	AA	Not Discussed	Custom-built	Not Discussed	4
[170]	No	smart contract	AA	Not Discussed	Not Discussed	Not Discussed	2
[109]	No	proxy re-encryption	Transfer	Consortium	Hyperledger Fabric	lightweight Proof-of-Work	3
[240]	No	homomorphic encryption	Transfer	Not Discussed	Not Discussed	Not Discussed	4
[162]	No	smart contract	AA	Not Discussed	Ethereum	PoW-infer	3
[241]	No	trusted execution environment	Storage	Not Discussed	Ethereum	PoW-infer	3
[242]	No	proxy re-encryption	Transfer	Private	Ethereum	PoW-infer	3
[243]	No	Identity Based Proxy Re-Encryption (IB-PRE)	Transfer	Consortium	Hyperledger Fabric	PBFT-infer	3
[149]	No	threshold signature	AA	Private	Hyperledger Besu	Istanbul Byzantine Fault Tolerant	4
[244]	No	multi-authority attribute-based encryption with verifiable outsourced decryption and hidden access policies	AA	Private	Ethereum	PoW-infer	2
[138]	No	PPSEB	search	Not Discussed	Not Discussed	Not Discussed	3
[245]	No	On-off chain storage	Storage	Private	Ethereum	PoW-infer	3
[167]	No	smart contract	AA	Private	Ethereum	PoW-infer	3
[246]	No	physical unclonable function	AA	Not Discussed	Hyperledger Fabric	PBFT-infer	3
[247]	No	fuzzy commitment	AA	Not Discussed	Not Discussed	Not Discussed	3
[248]	No	verifiable credential	AA	Not Discussed	hyperledger Indy	Not Discussed	3
[249]	No	On-off chain storage	Storage	Not Discussed	Ethereum	PoW-infer	3
[250]	No	access tree control lock scheme	AA	Not Discussed	Ethereum	PoW-infer	3
[185]	GDPR	On-off chain storage/proxy re-encryption	Storage; Transfer	Not Discussed	Hyperledger Fabric	PBFT-infer	3
[251]	No	proxy re-encryption	Transfer	Not Discussed	Not Discussed	Not Discussed	2
[252]	No	content extraction signature	AA	Not Discussed	Not Discussed	Byzantine fault-tolerant leader election scheme for Raft	3
[253]	No	heterogeneous signcryption with proxy re-encryption	AA	Not Discussed	Not Discussed	Not Discussed	2
[254]	No	attribute-based AA scheme with hidden policies	AA	Not Discussed	Not Discussed	Not Discussed	2
[166]	No	proxy re-encryption/smart contract	Transfer; AA	Private	Ethereum	Proof of authority	4
[255]	No	homomorphic encryption	Transfer	Private	Ethereum	PoW-infer	3
[256]	No	On-off chain storage	Storage	Not Discussed	Hyperledger fabric	PBFT-infer	3
[257]	No	Authentication role mapping	AA	Public	Binance smart chain	Proof of Stacked Authority	3
[258]	No	Attribute-Based Access control	AA	Not Discussed	Not Discussed	Not Discussed	2
[259]	No	K-nearest neighbour	AA	Not Discussed	Not Discussed	Not Discussed	2
[25]	GDPR	On-off chain storage	Storage	Public	Monero	Not Discussed	3
[260]	No	CP-ABE	AA	Not Discussed	Not Discussed	Not Discussed	2
[143]	No	Modular Order-Preserving Encryption	search	Not Discussed	Custom-built	Not Discussed	3
[261]	No	attribute-based access control	AA	Not Discussed	Hyperledger Fabric	Raft	3
[262]	No	quantum authentication	AA	Not Discussed	Custom-built	Not Discussed	2
[263]	No	homomorphic cryptosystem	Transfer	Not Discussed	Hyperledger fabric	PBFT-infer	4
[134]	No	pairing-based cryptography	Storage	Public	ethereum	PoW	3
[264]	No	CAViaR-based BSA	Transfer	Not Discussed	Not Discussed	Not Discussed	3
[265]	No	Smart contract	AA	Private	Not Discussed	Not Discussed	2
[266]	No	On/Off chain	Storage	Consortium	Hyperledger Fabric	Raft	4
[115]	No	On/Off chain; Smart contract	Storage; AA	Public	Ethereum	PoW	4
[139]	No	Searchable encryption; attribute-based encryption	Search; AA	Not Discussed	Ethereum	PoW	4
[267]	No	Garlic Routing	Transfer	Consortium	Not Discussed	Proof of epidemiology of interest (PoEoI)	3
[180]	No	On/Off chain; ABE	Storage; AA	Not Discussed	Hyperledger Fabric	Kafka	3
[268]	No	On/Off chain	Storage	Consortium	Not Discussed	Not Discussed	2
[173]	No	ABE	AA	Not Discussed	Ethereum	PoW-infer	2
[174]	No	ABE	AA	Not Discussed	Not Discussed	Not Discussed	2
[269]	No	PRE; On/Off chain	Transfer/Storage	Consortium	Not Discussed	Not Discussed	2
[270]	No	Smart contract	AA	Not Discussed	Hyperledger Fabric	PBFT-infer	2
[271]	No	Smart contract	AA	Private	Not Discussed	Not Discussed	4
[140]	No	inner product searchable encryption scheme with multi-keyword search	Search	Consortium	Not Discussed	PBFT	3
[272]	No	On/Off chain; Selective Sharing Mechanism	Storage; AA	Private	Hyperledger Fabric	PBFT	4
[273]	No	Lattice-Based Access Control (LBAC)	AA	Not Discussed	Ethereum	PoW-infer	3
[274]	No	On/Off chain; Smart contract	Storage; AA	Not Discussed	Ethereum	PoW-infer	3
[275]	No	PRE	Transfer	Hybrid	Ethereum	PoW-infer	3
[276]	No	Masked Authenticated Messaging (MAM)	Transfer	Not Discussed	IOTA Tangle	Markov Chain Monte Carlo (MCMC)	3
[130]	No	Hybrid Encryption (OK-HECCFHE)	Storage	Not Discussed	Not Discussed	Not Discussed	3
[68]	No	Smart contract; Proxy re-encryption	AA/Transfer	Public	Ethereum	PoW-infer	3
[277]	No	On/Off chain	Storage	Consortium	Hyperledger Fabric	Raft	4
[116]	No	Smart contract; Proxy re-encryption	AA/Transfer	Public	Ethereum	PoW-infer	4
[278]	No	Anonymous Ring Signatures	AA	Public	Not Discussed	Not Discussed	3
[111]	No	On/Off chain; Smart contract	Storage; AA	Public	Ethereum	PoW-infer	4
[279]	No	MA-RABE	AA	Not Discussed	Not Discussed	Not Discussed	3
[280]	No	On/Off chain; Smart contract	Storage; AA	Public	Ethereum	PoW-infer	4
[281]	No	PRE; Ring signature	Transfer/AA	Private	Ethereum	PoW-infer	3
[282]	No	Smart contract	AA	Private	Ethereum	PoW	3
[283]	No	On/Off chain; Smart contract	Storage; AA	Private	Ethereum	PoW-infer	3
[175]	No	CP-ABE and Smart contract; PRE	AA/Transfer	Consortium	Not Discussed	Not Discussed	2
[284]	No	On/Off chain	AA	Private	Not Discussed	Not Discussed	2
[285]	No	Lamport Merkle Authentication	AA	Private	Not Discussed	Not Discussed	2
[286]	No	Reputation-Based Cluster Head Selection	AA	Private	Not Discussed	Not Discussed	2
[98]	No	anomaly detection	AA	Consortium	Hyperledger Fabric	PBFT	4
[210]	No	CP-ABE	AA	Consortium	Hyperledger Fabric	Raft	4
[117]	No	Role-Based Access Control	AA	Public	Ethereum	PoS	3
[287]	No	Smart contract	AA	Private	Hyperledger Fabric	Raft	4
[288]	No	Fuzzy Attribute Set for Policy Obfuscation	AA	Consortium	Not Discussed	Not Discussed	2
[177]	No	CP-ABE; PRE	AA/Transfer	Private	Hyperledger Fabric	PBFT-infer	4
[164]	No	Smart contract; Proxy re-encryption	AA/Transfer	Private	Ethereum	PoW-infer	3
[289]	No	ABE	AA	Not Discussed	Not Discussed	Not Discussed	3
[118]	No	On/Off chain; Smart contract	Storage; AA	Public	Ethereum	PoW	3
[127]	No	Smart contract	AA	Public	Ethereum	PoW-infer	3
[290]	No	Smart contract	AA	Private	Ethereum	PoW-infer	3
[291]	No	Smart contract	AA	Not Discussed	Not Discussed	Not Discussed	3
[178]	No	CP-ABE	AA	Consortium	Not Discussed	Not Discussed	3
[292]	No	On/Off chain	Storage	Consortium	Hyperledger Fabric	Raft	3
[293]	No	Local Differential Privacy (LDP)	Transfer	Consortium	Hyperledger Fabric	PBFT-infer	3
[294]	No	Smart contract	AA	Not Discussed	Not Discussed	Not Discussed	2
[26]	Australian Privacy Principles (APPs); GDPR	On/Off chain	Storage	Consortium	Ethereum	PoW-infer	3
[295]	No	On/Off chain; Smart contract	Storage; AA	Consortium	Ethereum	Proof of Authority	3
[296]	No	Usage Control (UCON) Model	AA	Consortium	Hyperledger Fabric	PBFT-infer	3
[181]	No	CP-ABE & DAA	AA	Consortium	Hyperledger Fabric	BFT	3
[133]	No	Homomorphic Encryption	Storage	Not Discussed	Not Discussed	Not Discussed	3
[297]	No	zkp	AA	Consortium	Hyperledger Fabric	Raft	4
[298]	No	On/Off chain; Smart contract	Storage; AA	Consortium	Hyperledger Fabric	Kafka	3
[142]	No	CP-ABE; CP-ABSE	AA; Search	Consortium	Hyperledger Fabric	PBFT-infer	3
[299]	No	Mutual Authentication	AA	Not Discussed	Not Discussed	lightweight Proof of Work	2
[132]	No	Homomorphic Encryption	Storage	Consortium	Hyperledger Fabric	PBFT-infer	3
[99]	No	Smart contract	AA	Consortium	Hyperledger Fabric	PBFT	3
[100]	No	Mutual Authentication	AA	Consortium	Hyperledger Fabric	PBFT	3
[300]	No	Smart contract	AA	Consortium	Ethereum	PoW-infer	3
[301]	No	Smart contract	AA	Consortium	Hyperledger Fabric	PBFT-infer	3
[302]	No	Certificateless Aggregate Signcryption	AA	Consortium	Hyperledger Fabric	PBFT-infer	3
[303]	No	Smart contract	AA	Public	Ethereum	PoW-infer	3
[304]	No	ECMQV-MAC Protocol	AA	Consortium	Custom	Encrypted K-Means Clustering-based Stellar Consensus Protocol	3
[105]	No	Homomorphic Encryption	Transfer	Consortium	Hyperledger Fabric	PBFT	3
[119]	No	Smart contract	AA	Public	Ethereum	PoW-infer	3
[305]	No	privacy ontology	Storage	Not Discussed	Not Discussed	Not Discussed	2
[306]	No	Smart contract	AA	Consortium	Custom	PoW	3
[307]	No	Smart contract	AA	Consortium	Custom	Proof of Agreement	3
[120]	No	Smart contract	AA	Public	Ethereum	PoW	3
[308]	No	Token Shuffling	Storage	Not Discussed	Not Discussed	Not Discussed	2
[121]	No	Smart contract	Consent management	Public	Ethereum	PoW-infer	
[309]	No	Multi-authority Attribute-Based Access Control	AA	Not Discussed	Not Discussed	Not Discussed	2
[122]	No	Smart contract	AA	Public	Ethereum	PoW	3
[310]	No	FHE	Transfer	Consortium	Custom	Proof of Deep Learning	3
[311]	No	Smart contract	AA	Consortium	Hyperledger Fabric	PBFT-infer	3
[123]	No	Smart contract	AA	Public	Ethereum	PoW-infer	3
[179]	No	CP-ABE	AA	Consortium	Not Discussed	Not Discussed	3
[312]	No	Group Signature	AA	Consortium	Hyperledger Besu	PBFT	4
[101]	No	Double Signature Scheme	AA	Consortium	Hyperledger Fabric	PBFT	3
[313]	No	Self-Blindable Anonymous Authentication	AA	Consortium	Ethereum	Dynamic Proof-of-Work (dPoW) with checkpoints	3
[146]	No	Signcryption	Transfer	Not Discussed	Not Discussed	Not Discussed	3
[150]	No	CP-ABE; HE	AA; Verification	Private	Not Discussed	Not Discussed	3
[314]	No	On/Off chain	Storage	Public	Ethereum	PoW-infer	3
[144]	No	zkp	Verification	Consortium	Quorum	Raft-based	3
[315]	No	Multi-signature (Multisig) Contracts	AA	Consortium	Hyperledger Fabric	PBFT-infer	3
[316]	No	Chaincode	AA	Consortium	Hyperledger Fabric	Raft	3
[135]	No	PEKS	Search	Consortium	Not Discussed	Not Discussed	2
[317]	No	Proxy Re-encryption	Transfer	Consortium	Not Discussed	Not Discussed	3
[318]	No	Smart contract	AA	Consortium	Hyperledger Fabric	PBFT-infer	3
[319]	No	zkp	Verification	Consortium	Ethereum	ePoW	3
[182]	No	HE	Transfer	Not Discussed	Not Discussed	Not Discussed	2
[141]	No	Encrypted Data Search	Search	Consortium	Ethereum	PoW-infer	3
[320]	No	Dynamic Consent Management	AA	Consortium	Hyperledger Fabric	PBFT-infer	3
[321]	No	On/Off chain	Storage	Consortium	Hyperledger Fabric	PBFT-infer	3
[322]	No	Beacon Proxy	AA	Public	Ethereum	PoW-infer	3
[323]	No	ABE	AA	Not Discussed	Not Discussed	Not Discussed	3
[324]	No	CP-ABE; SE	AA; Search	Public - Infer	Ethereum	PoW - Infer	3
[325]	No	Authenticated Multi-Version Skip List	Search	Public - Infer	Ethereum	PoW - Infer	3
[326]	No	AES; Secondary Verification	Storage; AA	Consortium	Not Discussed	DPoS	3
[327]	No	Smart Contract	AA	Public - Infer	Ethereum	PoW - Infer	3
[328]	No	Quasi Sensitive Attribute Identification (QBSAI), Shuffled Random Starvation Link Encryption (SRSLE)	AA	Consortium	Hyperledger Fabric	PBFT - Infer	3
[329]	No	Role-Based Access Control	AA	Private	Hyperledger Fabric	BFT	3
[330]	Yes	Smart contract; on/off-chain	AA; Storage	Public	Ethereum	PoW/PoS	3
[331]	No	CP-ABE; ZKP	AA; Verification	Consortium	Not Discussed	Not Discussed	3
[332]	No	Smart contract	AA	Not Discussed	Not Discussed	Not Discussed	2
[333]	No	PRE	Transfer	Public	Ethereum	PoW - Infer	4
[334]	No	Smart contract	AA	Consortium	Hyperledger Fabric	Raft	3
[335]	Yes	smart contract	AA	Public - Infer	Ethereum	PoW - Infer	3
[336]	No	Differential Privacy	Transfer	Not Discussed	Not Discussed	Not Discussed	2
[337]	No	Smart contract	AA	Consortium	Rahasak	Not Discussed	3
[338]	No	Smart contract	AA	Public + Consortium	Ethereum	PoW - Infer	3
[339]	No	SE	Search	Public	Hyperledger Fabric	PBFT - Infer	4
[340]	No	zkp; PRE	Verification; Transfer	Consortium	Hyperledger Fabric	PBFT - Infer	3
[341]	No	2D Chaotic Mapping Encryption (2CME)	Storage	Consortium	Hyperledger Fabric	PBFT - Infer	4
[342]	No	CP-ABE; SE	AA; Search	Consortium	Not Discussed	Not Discussed	4
[343]	No	Smart contract	AA	Consortium	Not Discussed	Not Discussed	3
[344]	No	Smart contract	AA	Public - Infer	Ethereum	PoW - Infer	3
[345]	No	Smart contract; HE	AA; Storage	Public	Ethereum	PoW - Infer	3
[346]	No	zkp; smart contract	Verification; AA	Public	Ethereum	PoW - Infer	3
[347]	No	ABAC	AA	Consortium	Hyperledger Fabric	Raft	3
[348]	No	One-way Multi-hop Conditional Proxy Re-encryption (UMH-TIB-CPRE)	Transfer	Consortium	Not Discussed	DRK-PBFT (Dynamic Reputation K-means PBFT)	3
[349]	No	Smart contract	AA	Public	Ethereum	PoW - Infer	3
[350]	No	PRE	Transfer	Consortium	Hyperledger Fabric	PBFT - Infer	3
[351]	No	Smart contract	AA	Consortium	Hyperledger Fabric	Raft	4
[352]	No	Smart contract	AA	Consortium	Not Discussed	DPoS	3
[353]	No	Smart contract	AA	Public - Infer	Ethereum	PoW - Infer	3
[354]	No	Smart contract	AA	Not Discussed	Not Discussed	Not Discussed	2
[355]	No	Ciphertext-Policy Attribute-Based Encryption (CP-ABE)	AA	Consortium	Not Discussed	PBFT	3
[356]	No	Smart contract	AA	Public - Infer	Ethereum	PoW	3
[357]	No	Smart contract	AA	Public - Infer	Ethereum	Proof-of-Monitoring (PoM)	3
[338]	No	ZKP	Verification	Consortium - Infer	Hyperledger Fabric	PBFT - Infer	3
[66]	No	Smart contract	AA	Public	Ethereum	PoW - Infer	3
[358]	No	Modified Key Policy Attribute-Based Encryption (MKP-ABE)	AA	Private	Hyperledger Fabric	PBFT - Infer	3
[359]	No	ZKP	Verification	Consortium	Hyperledger Indy	RBFT - Infer	3
[360]	No	CP-ABE	AA	Public	Ethereum	PoW	3
[361]	No	Smart contract	AA	Consortium	Hyperledger Fabric	PBFT - Infer	3
[362]	No	Convergent Encryption	Storage	Private	Hyperledger Fabric	PBFT	3
[363]	No	ABE	AA	Consortium	Not Discussed	Not Discussed	3
[364]	No	PRE; Smart contract	Transfer; AA	Consortium	Hyperledger Fabric	Raft	4
[365]	No	BC-dPEKS; Smart contract	Search; AA	Consortium - Infer	Hyperledger Fabric	PBFT - Infer	3
[366]	No	On/Off chain storage; Smart contract	Storage; AA	Consortium - Infer	Hyperledger Iroha	YAC (Yet Another Consensus) - Infer	4
[367]	No	zkp	Verification	Not Discussed	Not Discussed	Not Discussed	2
[368]	No	CP-ABE; ZKP	AA; Verification	Consortium	Not Discussed	PBFT	3
[369]	No	Smart contract	AA	Consortium	Hyperledger Fabric	PBFT - Infer	3
[370]	No	PRE	Transfer	Consortium - Infer	Hyperledger Fabric	PBFT - Infer	3
[371]	No	Smart contract	AA	Consortium	Not Discussed	Pattern Proof of Malware Validation	3
[372]	No	PRE; Smart contract	Transfer; AA	Hybrid Blockchain	XuperChain	PBFT	3
[373]	No	Metadata-Based Privacy	Verification	Not Discussed	Custom Implementation	Proof of Authentication 2.0	3
[142]	No	CP-ABE with Hidden Policy; CP-ABSE	AA; Search	Consortium - Infer	Hyperledger Fabric	PBFT - Infer	3
[374]	No	Certificate-based Signcryption with Proxy Re-Encryption	Transfer	Not Discussed	Not Discussed	Improved DPoS	2
[375]	No	Smart contract	AA	Public	Not Discussed	PoW	3
[376]	No	Smart contract	AA	Public + Consortium	Hyperledger Fabric; Ethereum	BFT; PoW	3
[377]	No	Differential Privacy	Transfer	Consortium	Not Discussed	Committee-based Consensus	3
[378]	No	RBAC	AA	Consortium	Hyperledger Besu	Clique	3
[379]	No	Hybrid Chaotic Encryption	Storage	Public - Infer	Ethereum	PoW - Infer	3
[380]	No	ZKP	Verification	Private	Ethereum	PoW - Infer	3
[381]	No	CP-ABE	AA	Hybrid Blockchain	Not Discussed	Not Discussed	2
[382]	No	ABAC	AA	Consortium	Hyperledger Fabric	PBFT - Infer	4
[383]	No	On/Off chain; Smart contract	Storage; AA	Consortium	Hyperledger Fabric	PBFT - Infer	3
[384]	No	On/Off chain; Smart contract	Storage; AA	Consortium	Not Discussed	PBFT	3
[385]	No	On/Off chain; Smart contract	Storage; AA	Public	Ethereum	PoW - Infer	3
[386]	No	HE	Storage	Private	Not Discussed	Not Discussed	3
[387]	No	On/Off chain; Smart contract	Storage; AA	Consortium	Hyperledger fabric	PBFT - Infer	3
[388]	No	PRE	Transfer	Consortium	Hyperledger fabric	PBFT - Infer	3
[389]	No	zkp	verification	Not Discussed	Not Discussed	Not Discussed	2
[390]	No	Smart contract	AA	Public - Infer	Ethereum	PoW - Infer	3
[391]	No	Differential Privacy	Storage	Private	custom	Proof of Reputation	3
[392]	No	on/Off chain; Smart contract	Storage; AA	Private	Ethereum	PoW - Infer	3
[393]	No	ECC-based CP-ABE	AA	Public - Infer	Ethereum	PoW - Infer	3
[394]	No	Smart contrace	AA	Consortium	Hyperledger Fabric	PBFT - Infer	3
[395]	No	Certificateless Asymmetric Searchable Encryption	Search	Public - Infer	Ethereum	PoW - Infer	3
[396]	No	Smart contract	AA	Public - Infer	Ethereum	PoW - Infer	3
[397]	No	Hybrid Encryption (AES + Blowfish)	storage	Not Discussed	Not Discussed	Not Discussed	2
[398]	No	Smart contract	AA	Public - Infer	Ethereum	PoW - Infer	3
[399]	No	CP-ABE	AA	Consortium	Custom blockchain	Not Discussed	3
[400]	No	Threshold Proxy Re-encryption (TPRE)	Transfer	Consortium	Hyperledger Fabric	PBFT - Infer	3
[122]	No	Bloom Filters	Search	Not Discussed	Not Discussed	Not Discussed	2
[401]	No	Hybrid Encryption (AES+RSA)	Storage	Public - Infer	Ethereum	PoW - Infer	3
[402]	No	Smart contract	AA	Consortium	Hyperledger Fabric	PBFT - Infer	2
[403]	No	RBAC; On/Off chain	AA; storage	Consortium	Hyperledger Fabric	PBFT - Infer	3
[404]	No	RBAC	AA	Public - Infer	Ethereum	PoW - Infer	3
[405]	No	RBAC; On/Off chain	AA; storage	Consortium	Hyperledger Fabric	PBFT - Infer	3
[406]	No	RBAC; ZKP	AA;Verification	Public - Infer	Ethereum	PoW - Infer	3
[407]	No	Smart contract; zkp	AA; Verification	Public - Infer	Ethereum	PoW - Infer	3
[408]	No	PRE	Transfer	Not Discussed	Not Discussed	Not Discussed	2
[409]	No	On/Off chain	Storage	Not Discussed	Custom Blockchain	PoS	3
[410]	No	Onion Routing	Transfer	Not Discussed	Not Discussed	Not Discussed	2
[411]	No	Smart contract	AA	Public - Infer	Ethereum	PoW - Infer	3
[412]	No	Dual ElGamal Homomorphic Encryption (DEHE); Smart contract	Storage; AA	Public - Infer	Ethereum	PoW - Infer	3
[413]	No	zkp	Verification	Consortium	Hyperledger Indy	Plenum	2
[414]	No	Smart contract	AA	Public - Infer	Ethereum	PoW - Infer	3
[415]	No	Smart contract; On/Off-chain	AA; Storage	Consortium	Hyperledger Fabric	PBFT - Infer	3

## Footnotes

1 https://www.hyperledger.org/use/fabric
